# Occurrence, Bioaccumulation, Metabolism and Ecotoxicity of Fluoroquinolones in the Aquatic Environment: A Review

**DOI:** 10.3390/toxics11120966

**Published:** 2023-11-29

**Authors:** Mengnan Shen, Yi Hu, Ke Zhao, Chenyang Li, Binshuo Liu, Ming Li, Chen Lyu, Lei Sun, Shuang Zhong

**Affiliations:** 1 Key Laboratory of Songliao Aquatic Environment, Ministry of Education, Jilin Jianzhu University, Changchun 130118, China; smn930@aliyun.com (M.S.); 13527431798@163.com (Y.H.); zhaoke326@126.com (K.Z.); lichenyang0331@126.com (C.L.); liubinshuo73@163.com (B.L.); mgvi@163.com (M.L.); chen_327@hotmail.com (C.L.); 2 Liaoning Provincial Mineral Exploration Institute Co., Ltd., Shenyang 110031, China; 3 Key Laboratory of Groundwater Resources and Environment, Ministry of Education, Jilin University, Changchun 130021, China

**Keywords:** antibiotic, environmental concentration, bioaccumulation, metabolism, ecotoxicology

## Abstract

In recent years, there has been growing concern about antibiotic contamination in water bodies, particularly the widespread presence of fluoroquinolones (FQs), which pose a serious threat to ecosystems due to their extensive use and the phenomenon of “pseudo-persistence”. This article provides a comprehensive review of the literature on FQs in water bodies, summarizing and analyzing contamination levels of FQs in global surface water over the past three years, as well as the bioaccumulation and metabolism patterns of FQs in aquatic organisms, their ecological toxicity, and the influencing factors. The results show that FQs contamination is widespread in surface water across the surveyed 32 countries, with ciprofloxacin and norfloxacin being the most heavy contaminants. Furthermore, contamination levels are generally higher in developing and developed countries. It has been observed that compound types, species, and environmental factors influence the bioaccumulation, metabolism, and toxicity of FQs in aquatic organisms. FQs tend to accumulate more in organisms with higher lipid content, and toxicity experiments have shown that FQs exhibit the highest toxicity to bacteria and the weakest toxicity to mollusk. This article summarizes and analyzes the current research status and shortcomings of FQs, providing guidance and theoretical support for future research directions.

## 1. Introduction

In recent years, antibiotics have played a pivotal role in controlling bacterial infections, reducing mortality rates, and extending human lifespans. Consequently, antibiotics have gained widespread application across medical, pharmaceutical, and livestock sectors [1,2]. Studies have shown that upon entering the body, antibiotics undergo incomplete absorption, with almost 70% being excreted as either parent compounds or metabolites [3]. Consequently, these antibiotics continually infiltrate aquatic environments through wastewater discharges and surface runoff, establishing a “pseudo-persistent” state [4]. As a result, organisms inhabiting marine ecosystems may be subjected to long-term drug exposure. Fluoroquinolones (FQs) represent a class of chemically synthesized antibacterial drugs used for the treatment of both Gram-negative and Gram-positive bacteria. Their physicochemical properties are shown in Table S1 [5,6,7,8,9]. Due to their broad spectrum of antimicrobial activity, strong bactericidal ability, high oral absorption efficiency, and lack of cross-resistance with other antibacterial drugs [10,11] FQs have found extensive use in medical, livestock, and aquaculture industries [2,12]. Therefore, FQs have emerged as one of the primary residual antibiotics in aquatic environments.

Currently, researchers have detected nearly 10 residues of FQs in surface waters of China, Spain, Brazil, Malaysia, and Kenya, with concentrations ranging from ng/L to μg/L. Notably, surface water near Juja in Kenya has been found to have high residue concentrations of ciprofloxacin (CIP) (75.70 μg/L) and norfloxacin (NOR) (52.60 μg/L) [4,5,13,14,15,16]. FQs in water can enter organisms through bioaccumulation. Significant residues of FQs have been detected in various aquatic organisms, such as phytoplankton, zooplankton, zoobenthos shrimp, and fish (ranging from non-detectable (ND) to 342 ng/g dry weight (dw)) [4]. Previous studies have shown that FQs can form multiple metabolites in organisms under the catalysis of relevant enzymes [17,18]. Both the parent compounds and metabolites of FQs may have toxic effects on organisms [19]. Therefore, it is necessary to further investigate the occurrence, bioaccumulation, metabolism, and toxicity of FQs in aquatic environments to determine their ecological risks. Currently, there have been several studies that have provided a comprehensive review of the occurrence and biological toxicity of FQs in aquatic environments [20]. These studies have analyzed the impact of FQs on ecosystems and human health from the perspectives of national income, environmental conditions, and synergistic effects with other pollutants [20,21,22,23]. However, these studies mainly focus on the pollution status of FQs reported before 2020, with limited discussions on the accumulation and metabolism patterns of FQs in aquatic organisms. Therefore, it is necessary to further investigate the latest pollution status of FQs in aquatic environments, especially in terms of accumulation, metabolism, and toxicological impacts, in order to assess their ecological risks by monitoring their long-term pollution status.

This study aimed to compile and analyze the concentrations of 15 frequently encountered FQs in global surface water since 2020. The FQs included CIP, ofloxacin (OFL), NOR, enrofloxacin (ENR), lomefloxacin (LOM), danofloxacin (DAN), pefloxacin (PEF), fleroxacin (FLE), marbofloxacin (MAR), sarafloxacin (SAR), enoxacin (ENO), difloxacin (DIF), levofloxacin (LEV), moxifloxacin (MOX), and flumequine (FLU). At the same time, this study explored and summarized the bioaccumulation, metabolism, biological toxicity, and influencing factors of these antibiotics in organisms.

## 2. The Pollution Status of FQs in Surface Water

**Figure 1:**
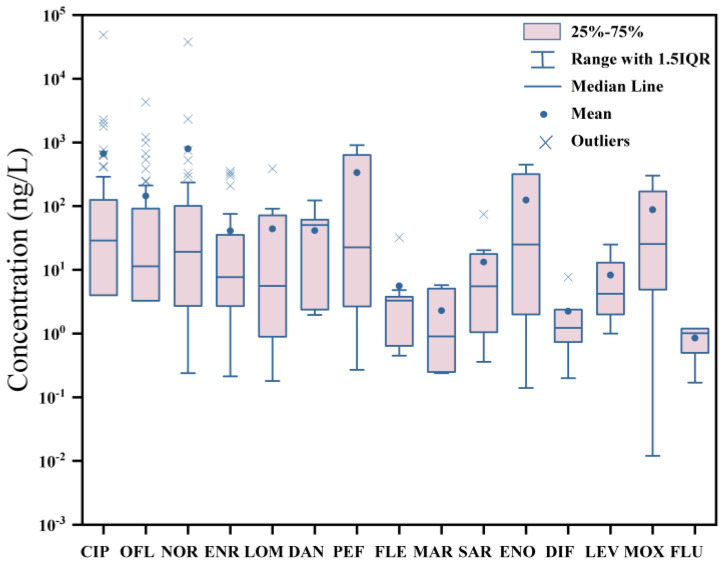
Box–and–whisker plot showing the mean concentrations of detected antibiotics globally in surface waters. This plot shows the mean concentrations of 15 FQs listed in Table S2.

**Figure 2:**
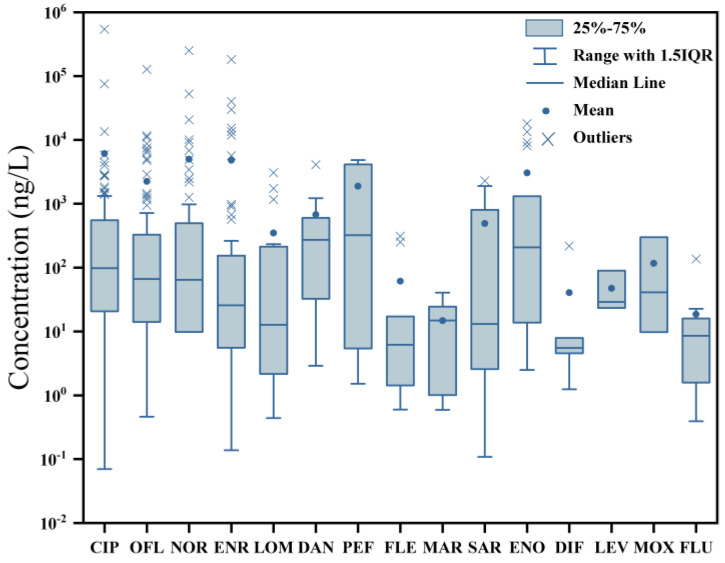
Box–and–whisker plot illustrating the maximum concentrations of detected antibiotics globally in surface waters. This plot shows the maximum concentrations of 15 FQs listed in Table S2.

In this study, a search was conducted in the Web of Science database (http://www.webofknowledge.com/, accessed on 31 August 2023) using the keywords “fluoroquinolones occurrence surface water” to retrieve relevant literature. Publications from 2020 to 2023 were collected. The concentrations of 15 common FQs (CIP, OFL, NOR, ENR, LOM, DAN, PEF, FLE, MAR, SAR, ENO, DIF, LEV, MOX, and FLU) mentioned in the literature were compiled in Table S2. Based on these data, the average and maximum concentrations of the FQs were plotted in Figure 1 and Figure 2, respectively. When multiple values were reported in the literature, the mean value was given priority, followed by the median value. If the mean or median value was not provided in the publication, it was calculated using the raw data to ensure equal weight for each study in the graph. For values below the limit of quantification (LOQ), half of the LOQ value reported in the corresponding literature was used for calculation [24].

This study documented the concentration distribution of 15 FQs in surface water from 32 countries (Table 1 and Table S2). According to the “Human Development Report” published by the United Nations in 2020, this study classifies the countries mentioned in the literature into two categories developing countries and developed countries to facilitate research analysis [25]. Significant variations in antibiotic concentrations were observed between different countries and regions, with generally higher FQs levels found in surface water from developing countries compared to developed ones. Notably, surface water in India displayed high concentrations of CIP at 542.45 μg/L [26] and Kenya at 75.70 μg/L [16]. The Mediterranean waters of Tunisia exhibited elevated levels of ENR (20.70 μg/L) and NOR (40.20 μg/L) [27]. Additionally, FQs were detected in surface water from developing countries such as Turkey [28], Bangladesh [29], Brazil [30], and China [31,32,33,34,35,36,37,38], with average concentrations in the tens of μg/L. In contrast, lower FQs concentrations were detected in surface water from developed countries, peaking at only a few μg/L. For instance, in the Charmoise River in France, the maximum concentrations of CIP and OFL were 1.52 μg/L and 2.89 μg/L, respectively [39]. Moving to North Carolina, USA, the highest concentration of DAN was found to be 1.23 μg/L [40]. Shifting focus to the five most frequently mentioned FQs in the literature (CIP, OFL, NOR, ENR, and LOM), an examination was conducted on the number of countries reporting maximum antibiotic concentrations in surface water reaching μg/L levels. The findings revealed that the proportion of developing countries exceeded that of developed countries, with percentages of 63.16% (CIP), 62.50% (OFL), 91.67% (NOR), 100% (ENR), and 100% (LOM). Furthermore, the presence of CIP has only been reported in surface water in countries such as Bangladesh [29], Australia [41], and Pakistan [42], while in Switzerland [42], only the presence of NOR has been reported. Meanwhile, China has reported the presence of all types of antibiotics in surface water. This study provides a comprehensive overview of the primary sources of FQs in different regions and water bodies. FQs contamination in water bodies can be attributed to three main factors. Firstly, medical wastewater, which includes the discharge of wastewater containing FQs residues from medical institutions and patients. Secondly, agriculture and aquaculture, where FQs are extensively used for preventing and treating animal infections, potentially leading to their entry into the environment through aqua-culture wastewater and agricultural irrigation water. Thirdly, discharge from wastewater treatment plants, responsible for handling the treatment of wastewater from urban and industrial areas. However, it is worth noting that complete FQs removal during the treatment process may not always be achieved, resulting in the presence of drug residues in the effluent that can enter the water environment [26,35,37]. Gao et al. [8] found that in the Liaohe River Basin in China, the main sources of FQs contamination were wastewater discharge from wastewater treatment plants and agricultural aquaculture. Another study identified wastewater discharge from the pharmaceutical industry in India as the main factor causing FQs pollution in surface water [43]. It is worth noting that regional differences observed are likely due to variations in locality used antibiotics, and the data may also vary due to regional preferences in detecting certain antibiotics. Therefore, the absence of reported antibiotics in surface water does not necessarily imply their absence in a particular region.

A statistical analysis was conducted on the average and maximum concentrations of the selected 15 FQs in global surface water. The study found that the 50th percentile of the average concentration of these FQs ranged from 0.91 to 50.60 ng/L (Figure 1), while the 50th percentile of the maximum concentration ranged from 5.53 to 323.00 ng/L (Figure 2). Notably, PEF and MAR were the only FQs differing by one order of magnitude between their average and maximum concentrations. Additionally, NOR exhibited the highest mean average concentration (795.00 ng/L), closely followed by CIP (669.10 ng/L). In terms of maximum concentrations, CIP took the lead at 542.45 μg/L, followed by NOR at 251.14 μg/L. These findings underscore a significant level of contamination in global surface water due to FQs, especially CIP and NOR. Therefore, addressing FQ pollution in water environments warrants extensive attention.

## 3. Bioaccumulation of FQs in Aquatic Organisms

In recent years, with the extensive use of FQs, substantial amounts of these compounds have been detected not only in surface waters worldwide but also in the tissues of aquatic organisms, such as fish, crabs, oysters, shrimp, and phytoplankton (Table 2). The main FQs detected included CIP, OFL, NOR, ENR, ENO, LOM, PEF, MAR, and SAR. The results revealed that CIP had the highest detection frequency in the tissues of organisms, while LOM exhibited the highest enrichment content. For instance, in China’s Taihu Lake, the residual amount of CIP in bivalves (12.00 to 80.00 ng/g dw) was significantly higher than in phytoplankton (ND to 30.00 ng/g dw) [4]. Similarly, in the Beibu Gulf of China, the average concentration of NOR in crab tissues exceeded that of ENR by 10.80 times [1]. Consequently, researchers conducted extensive studies on the bioaccumulation patterns and influencing factors of FQs in aquatic organisms.

### 3.1. The Bioaccumulation Pattern of FQs in Aquatic Organisms

#### 3.1.1. Bioaccumulation of Different FQs

Understanding the bioaccumulation pattern of pollutants is crucial for accurately assessing their ecological health risks. Researchers, through extensive laboratory simulation experiments, found that different FQs exhibit varying bioaccumulation patterns within the same organism [110,111,112,113]. For instance, after exposing *Cyprinus carpio* to 8 different FQs (Balofloxacin (BAL), ENO, ENR, FLE, LOM, MOX, OFL, and Sparfloxacin (SPA)) for 28 days, the concentration of MOX in fish tissues significantly surpassed other FQs. The fish’s liver showed the highest bioaccumulation of MOX, reaching 42.94 times, 35.59 times, and 34.23 times higher than OFL, LOM, and SPA, respectively [110]. Similarly, Chen et al. [112] discovered that the bioaccumulation ability of FLE and DIF in aquatic plants was significantly greater than that of OFL and MAR. However, the underlying reasons for such differences in bioaccumulation ability have yet to be determined. Chen et al. [112] and Claude et al. [114] proposed a positive correlation between the bioaccumulation concentration of compounds and their log Kow values. Simultaneously, Zhou et al. [115] and Zhang et al. [116] found a negative correlation between log bioconcentration factor (BCF) values in aquatic animals and log Kow. However, the distribution coefficient (log D) associated with pH values is a better predictor of compound bioaccumulation within organisms. Furthermore, other studies have revealed that factors such as the substituent interaction between R_7_ and R_8_ positions in the FQs structure (Figure S1), as well as the compound’s chemical structure, solubility, and molecular weight, may also impact their accumulation ability within organisms [110,113].

#### 3.1.2. Bioaccumulation of FQs in Different Organisms

Different organisms exhibit significantly varied capabilities in the enrichment of FQs, indicating species-specific bioaccumulation of these compounds. Zhang et al. [116] conducted a study to detect the residual concentrations of ∑FQs in fish, shrimp, and *Stichopus japonicus*. Their findings revealed that FQ concentrations in fish (0.61 to 171.00 ng/g ww) were significantly higher than those in shrimp (0.32 to 27.30 ng/g ww) and *S. japonicus* (0.39 to 1.19 ng/g ww). Moreover, in aquatic plants, Chen et al. [112] observed that *Cyperus papyrus* exhibited significantly higher bioaccumulation abilities for five FQs (PEF, MAR, OFL, FLE, DIF) compared to *Lythrum salicaria*, *Ruellia simplex*, and *Acorus calamus*. The reasons for these bioaccumulation differences may be attributed to the organisms’ capabilities in antibiotic uptake, metabolism, as well as the content of proteins and lipids within their bodies [17,116,117]. Additionally, the differential bioaccumulation abilities of aquatic plants for FQs might be associated with the activity of their root microbiota [118]. It is worth noting that current research on the enrichment of FQs mainly focuses on different animals and plants, with limited studies on the differences in bioaccumulation among different trophic levels within ecosystems. Further research in this area is warranted.

#### 3.1.3. Bioaccumulation of FQs in Different Tissues and Growth Stages

Further research has revealed that different tissues and organs in organisms exhibit varying capabilities to accumulate FQs. Sun et al. [119] and Chen et al. [110] explored the distribution of FQs in various tissues of *C. carpio*, revealing the liver as the central organ of bioaccumulation, with a proportion as high as 70.99% (MOX). Similar findings were observed in *Danio rerio* [111] and *Bellamya aeruginosa* [120], where the OFL content in the viscera was significantly higher than in other tissues. This distribution difference may be related to the phospholipid content in organisms, as phospholipids, the main components of biological cell membranes, are widely distributed in visceral tissues such as the liver and kidneys [121]. Given the lipophilic nature of the majority of FQs, they are more prone to bioaccumulate in tissues with higher lipid content. Similar studies have identified a significant positive correlation between the bioaccumulation of FQs and lipid content in organisms at different growth stages (*Eichhornia crassipes*, fish, shrimp, and *S. japonicus*) [116,122]. In addition, Zhu et al. [17] observed that the distribution proportion of ENR in *S. japonicus’* body wall and mouth increases with exposure time, while the gastrointestinal and respiratory tracts decrease with exposure time. Therefore, the distribution of antibiotics in various tissues of organisms may also be related to the metabolic capacity of different tissues at different periods. In conclusion, while phospholipids are an essential factor influencing the distribution of FQs in organisms, factors such as tissue metabolism capacity can also impact their distribution within the body.

When exploring the distribution of FQs within aquatic plants, it has been observed that roots serve as the primary sites for bioaccumulation [112,113,122,123,124]. For example, Yan et al. [125] and Liu et al. [113] exposed *E. crassipes* and *Phragmites australis* to CIP-contaminated water, revealing that CIP concentration in the root tissues exceeded that in stems and leaves by 1 to 2 orders of magnitude across various exposure levels. Through transpiration, antibiotics accumulated in the roots are transported to stem and leaf tissues. The distance of this transport, influenced by photosynthetic activity, influences the concentration of antibiotics in stem and leaf tissues and the ability of self-migration and transformation [122,126].

#### 3.1.4. Bioaccumulation of FQs in Different Exposure Concentration and Duration

In general, the bioaccumulation of pollutants in organisms is directly proportional to the exposure concentration, while the BCF shows an inverse relationship. For example, Deng et al. [127] observed a 2.79-fold increase in CIP content in the roots of *E. crassipes* when exposed to 1000 μg/L CIP (8.56 μg/g) for 7 days compared to 10 μg/L CIP (3.01 μg/g). He et al. [120] investigated NOR and OFL content in the muscular foot of the *B. aeruginosa* after 28 days of exposure. They found that the NOR (20.68 ng/g) and OFL (94.38 ng/g) levels in the 2 μg/L group were only 0.84% and 3.60% of those in the 2000 μg/L group, respectively. BCF values in the 2 μg/L group were 10.34 L/kg for NOR and 47.19 L/kg for OFL, while values in the 2000 μg/L group exceeded 5 L/kg. The higher bioaccumulation of antibiotics at higher concentrations may be due to their passive transport within organisms. Simultaneously, higher antibiotic concentrations can induce lipid peroxidation in cell membranes, limiting their ability to be consumed and transferred within the organisms and resulting in reduced BCF values [128,129]. Regarding the influence of exposure time, studies on organisms such as the *B. aeruginosa* [120], *C. carpio* [110], *E. crassipes* [123], and *S. japonicus* [17] revealed an increasing trend in FQ content with prolonged exposure time. However, beyond a certain threshold, the FQ content in organisms exhibited a fluctuating pattern. The fluctuation pattern observed can be attributed to the metabolic processes and excretion mechanisms of organisms in response to pollutants [110,130]. When the rate of absorption of FQs exceeds the rate of metabolism and excretion, the concentration of FQs in the organism gradually accumulates. However, as the metabolic and excretion processes strengthen, it can lead to a decrease in the concentration of FQs in the organism. Once the concentration of FQs reaches a certain low point, FQs present in the water can be reabsorbed, resulting in a subsequent increase in the concentration of FQs in the organisms. This periodic process of metabolism and excretion accounts for the fluctuation pattern observed in the concentration of FQs in organisms.

### 3.2. The Impact of Other Factors on the Bioaccumulation of FQs in Aquatic Organisms

#### 3.2.1. Effects of Coexisting Pollutants

In aquatic environments, the coexistence of multiple pollutants often leads to complex pollution, an essential factor affecting the bioaccumulation of FQs in marine organisms. For example, Zhao et al. [111] found that the addition of copper (Cu) can promote the uptake of ENR and OFL in *D. rerio*, and the promotion effect is more significant at low Cu concentration (2.56 μg/L) compared to high Cu concentration (25.6 μg/L). Marcelo et al. [131] studied the bioaccumulation of multiple antibiotics (amoxicillin, ENR, and doxycycline) in *Lemna minor*. They found that compared to a single exposure, the concentration of ENR in *L. minor* decreased by 24.80% to 37.50% after binary or ternary mixture exposure. The decrease in concentration may result from the competition for adsorption sites on the surface of the biofilms by multiple antibiotics. In addition, it has been found that dissolved organic matter (DOM) can not only compete for adsorption sites on the biofilm surface, thereby reducing the bioavailability of FQs [132], but also form complexes with pollutants through chelation reactions, further reducing their bioavailability [133]. Therefore, the presence of DOM may also reduce the absorption of FQs by organisms.

#### 3.2.2. Effects of Environmental Factors

Furthermore, changes in environmental factors can also influence the bioaccumulation of FQs in organisms. Studies have shown that FQs are ionizable compounds, with their cationic and anionic parts facing challenges in passing through cell membranes due to electro-repulsion, electro-attraction, and ion trapping effects. In contrast, the non-ionized molecular part can undergo ‘ion trapping’, becoming trapped inside the cell membrane and facilitating the absorption of the compound’s zwitterionic form by plants [134]. Therefore, the water’s pH can play a role in influencing the bioaccumulation of FQs in organisms. On the other hand, sediment particles in water can adsorb antibiotics, thus reducing their bioavailability [135]. Changes in water salinity also contribute to alterations in the distribution of antibiotics between the water phase and solid phase, affecting their bioaccumulation [135]. However, there are currently no reports on the impact of environmental factors on the bioaccumulation of FQs in organisms. Therefore, further research is warranted to investigate this aspect and gain a more in-depth understanding.

## 4. Metabolism and Half-Life of FQs in Aquatic Organisms

Exogenous compounds entering the organism undergo biotransformation under the action of relevant metabolic enzymes [24]. The metabolism of these compounds in the organism is mainly divided into three phases. In Phase I, hydrolysis, oxidation, or reduction reactions take place under the catalysis of Phase I enzymes such as CYP450 enzymes and peroxidases, resulting in the formation of more hydrophilic compounds [136,137]. Moving into Phase II, subsequent to Phase I, enzymes like glutathione-S-transferases, methyltransferases, and transaminases catalyze the opening of the ring, leading to the formation of small molecular compounds [138,139]. Finally, Phase III is a distinctive metabolic stage in plants that involves the separation and storage of the metabolic products within the cell wall or vacuoles [124,140,141].

Currently, researchers have detected metabolites of different FQs, including ENR, CIP, LEV, MOX, and GAT (Figure S2), in aquatic plants (*Chlamydomonas reinhardtii* [18], *Chlorella vulgaris* [139,142], *Scenedesmus obliquus* [139,143], *E. crassipes* [122,123], *Chrysopogon zizanioides* [136], *Oryza sativa* L. [141]) and aquatic animals (*S. japonicus* [17], *Sparus aurata* [144], *Dicentrarchus labrax* [145]). In aquatic plants, the primary metabolic pathways for FQs involve ring cleavage and hydroxylation of the piperazine ring. In algae, over 40% of the metabolites from FQs metabolism are formed through ring opening, followed by hydroxylation, dealkylation, demethylation, and oxidation. Similarly, in aquatic animals, ring cleavage and hydroxylation of the piperazine ring remain the main metabolic pathways for FQs. However, unlike plants, defluorination is a significant pathway for metabolite formation in almost all aquatic animals, particularly in *S. japonicus* [17], where 80% of ENR metabolites are formed through defluorination. Thus, the primary metabolic pathways of FQs in aquatic organisms include ring cleavage, hydroxylation, and defluorination.

Research has revealed variations in both the quantity and composition of FQ metabolites across different tissues and organs in aquatic organisms. Saumik et al. [136] identified ten metabolites of CIP in *C. zizanioides*, with two in the roots and nine in the stems. Hu et al. [141], detected six CIP metabolites in *Oryza sativa* L., while only very few were detected in the stems (two) and leaves (one). A study on the *S. japonicus*, a marine organism, found that ENR formed five metabolites in its body, with the lowest concentration of parent compounds observed in the digestive tract and the highest concentration of metabolites [17], indicating that the *S. japonicus’* digestive tract is the primary site for ENR metabolism. The metabolism of antibiotics primarily occurs under the catalysis of specific enzymes, so the expression and activity of different metabolic enzymes may cause differences in FQs metabolism among other tissues of organisms. It is important to note that certain phase I metabolites can be as toxic as, or even more toxic than, the parent compounds [124,140]. For example, Hossein et al. [19] found that the metabolite of CIP exhibited significantly lower half-lethal concentrations (EC_50_) than the parent compound for fish, daphnids, and green algae. Therefore, further research is necessary to investigate the environmental hazards posed by FQs metabolites.

Concurrently, studies have explored the half-life of FQs in aquatic organisms, revealing how factors like compound type, species, and tissue distinctions influence this duration. For instance, Chen et al. [110] conducted a 28-day exposure of *C. carpio* to different FQs in water. During the subsequent 96-h elimination period, the concentration of SPA in the liver decreased by nearly 90%, while ENR remained at 96.40%. In another study, Song et al. [146] found that the half-life of DAN in *C. carpio haematopterus* bile tissue (170.24 h) was significantly longer than in muscle plus skin (47.89 h) and plasma (59.11 h). Furthermore, Wang et al. [147] discovered that the half-life of NOR in the kidney of *Sparus macrocephalus* (3.87 days) was almost double that of Japanese sea perch. These findings highlight the complex interplay of compound characteristics and biological factors in determining the persistence of FQs in aquatic environments. Currently, there is a dearth of information regarding the half-life of FQ in aquatic plants. Consequently, it is necessary to conduct additional research to facilitate a more comprehensive understanding of this phenomenon.

## 5. Toxicity of FQs

### 5.1. EC50 Values of FQs

**Figure 3:**
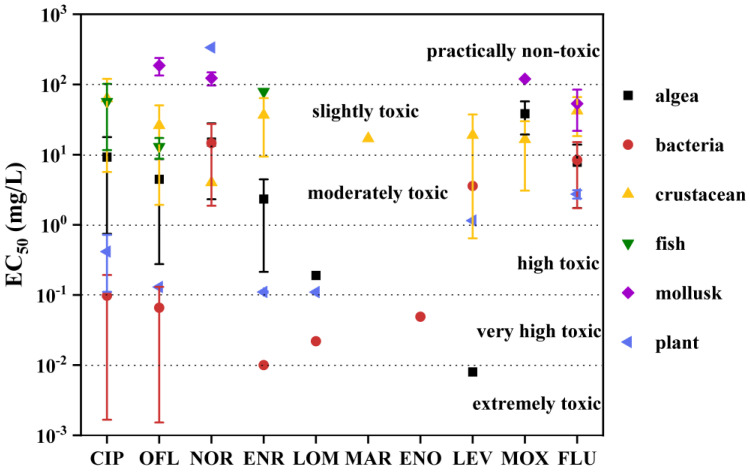
Ecotoxicity of 10 FQs in different groups of organisms. The figure shows the mean average value of EC_50_ for the same group of organisms, and the error bar represents the standard deviation.

This study assessed the toxicity of 10 FQs in algae, bacteria, crustaceans, fish, mollusk, and plants (Figure 3 and Table S3) [19,28,120,148,149,150,151,152,153,154,155,156,157,158,159,160,161,162,163,164,165,166,167,168,169,170,171,172,173,174,175,176,177,178,179,180,181]. According to the classification criteria proposed by the Joint Group of Experts on Scientific Aspects of Marine Environmental Protection (GESAMP) [182], the toxicity of FQs was categorized (Figure 3), showcasing a variance of 1 to 4 toxicity levels among different aquatic organisms. As noted by Pavla et al. [28], distinct species exhibit varying degrees of toxicity in response to antibiotics. Bacteria proved susceptible to FQs, with EC_50_ values ranging from 0.01 to 23.60 mg/L. Among them, CIP, OFL, ENR, LOM, and ENO exhibited very high toxicity to bacteria, as their average EC_50_ values fell below 10^−1^ mg/L. Algae and plants demonstrated the next tier of sensitivity, with 71.43% (algae) and 57.14% (plants) of the tested FQs classified as moderately toxic or higher. Notably, LEV exhibited extremely toxic effects on *M. aeruginosa* (24 h), with an EC_50_ value of 0.008 mg/L [154]. Conversely, crustaceans, fish, and mollusk showed relatively weaker sensitivity to FQs. Among them, mollusk displayed the least sensitivity, with EC_50_ values ranging from 31.10 to 222.60 mg/L, and almost 75% of the tested FQs demonstrated negligible toxicity to this species [120,176,181]. Fish and crustaceans exhibited mildly toxic effects, with EC_50_ values spanning from 2.17 to 192.00 mg/L.

Comparing the toxicity of various FQs to aquatic organisms revealed that MOX, NOR, and FLU exhibit relatively weak toxicity. NOR proved non-toxic to both plants and mollusk, with EC_50_ values ranging from 104.50 to 336.00 mg/L [120,168]. MOX similarly showed non-toxicity to mollusk, with an EC_50_ of 120 mg/L [176] (Table S3). Regarding the other FQs, at least one showed high toxicity to aquatic organisms, reaching levels classified as “high toxic” or higher. Assessing the percentage of species tested with a toxicity level classified as high or above, LOM (100%) and ENO (100%) demonstrated the highest toxicity, followed by CIP (40%) and ENR (40%). It should be noted that the experimental conditions, such as the developmental stage of organisms, water pH, temperature, light conditions, etc., were not considered in the analysis of the collected aquatic organism samples. For instance, FQs are ionizable compounds, and changes in water pH may affect their ecotoxicity [183,184]. In addition, conclusions could not be drawn for some FQs (such as DAN, PEF, FLE, SAR, and DIF) due to a lack of toxicity data.

### 5.2. Toxicological Effects of FQs on Aquatic Organisms

The toxic effects of FQs on aquatic organisms primarily involve three aspects: (1) the antioxidant defense system, including the concentrations of hydrogen peroxide (H_2_O_2_), malondialdehyde (MDA), glutathione (GSH), glutathione S-transferase (GST), and glutathione peroxidase (Gpx), as well as activities of superoxide dismutase (SOD), catalase (CAT), peroxidase (POD), and ascorbate peroxidase (APX); (2) the growth, development, and behavioral activities of organisms; and (3) genetic damage and genetic toxicity.

Research has found that FQs have an impact on the antioxidant defense system and growth development of aquatic plants. FQs can induce the production of reactive oxygen species (ROS) within organisms. The bioaccumulation of these ROS can aggravate lipid peroxidation in cell membranes, leading to cell membrane rupture, damage, and even apoptosis [185,186]. H_2_O_2_, a type of ROS, exhibited a substantial increase in *L. minor* exposed to CIP, with the 1.05 mg/L exposure group showing nearly three times the content of the control group, as observed by Marcelo et al. [129]. Another common ROS, O^2−^, undergoes peroxidation reactions with cell membrane lipids, generating oxidative products like MDA. Therefore, MDA levels indirectly reflect the severity of ROS attack on the organism [187]. Researchers exposed the *Chlamydomonas mexicana* [188] and the *Myriophyllum verticillatum* [189] to different FQs. The results revealed a significant rise in MDA levels in organisms exposed to high concentrations of FQs, indicating pronounced damage to cell membranes caused by ROS. Within organisms, enzymes such as SOD, CAT, POD, and APX play roles in eliminating O^2−^ and H_2_O_2_, with their activity levels reflecting the organism’s intermittent capacity to remove ROS. In a study by Nie et al. [190], *Pseudokirchneriella subcapitata* was exposed to CIP (0 to 2.5 mg/L), and after 96 h, the activities of SOD, CAT, and APX were measured. The results showed that as the exposure concentration increased, SOD activity increased, while the activities of CAT and APX exhibited a trend of initially low promotion and inhibition. Meanwhile, in organisms such as *Prorocentrum lima* and *Chlorella* sp (NOR) [191], *L. minor* (OFL) [192], *E. crassipes* (CIP) [127], and *P. australis* (CIP) [113], activities of SOD, CAT, POD, and APX in the presence of FQs were higher than those in the control group. However, under high concentrations or prolonged exposure, the activities of these enzymes would decrease, indicating that low FQ concentrations can trigger enzyme production for ROS elimination. As the oxidation level increases, the organism’s capacity to produce specific enzymes to eliminate ROS diminishes.

FQs in water can also have an impact on the growth and development of aquatic plants. Studies have shown that FQs can hinder photosynthesis in algae (*C. vulgaris* [193] and *Scenedesmus dimorphus* [174]) and aquatic plants (*L. minor* [129] and *E. crassipes* [125]) by disrupting the thylakoid membrane and inhibiting the expression of critical enzymes in the photosynthetic electron transport chain. Hong et al. [189] noted a positive correlation between the exposure concentration of ENR and the proportion of yellow leaves in *M. verticillatum*, with a 29.03% increase in the proportion of yellow leaves under 50 mg/L ENR exposure. At the molecular level, heightened concentrations of FQ (ENR) not only increase the transcription levels of genes related to photosynthesis in *Chlorella pyrenoidosa* (psaB and psbC) [169], but also inhibit chloroplast-specific enzyme (DNA gyrases) activity in plants [113].

For aquatic animals, FQs can also influence their antioxidant defense system, growth and development, behavior, and genetic integrity. Researchers conducted experiments on *Ctenopharyngodon idellus* (ENR) [186], *D. rerio* (CIP) [194], and *Pseudosciaena crocea* (NOR) [195], exposing them to various FQs. The findings revealed a significant increase in the MDA content within aquatic organisms’ bodies when exposed to high concentrations of FQs. Additionally, other studies demonstrated that FQs in water can enhance the activity of SOD and CAT in *Cirrhinus mrigala* (CIP) [196] and *D. rerio* (NOR) [197]. Investigation into enzyme changes associated with organism metabolism further showed a notable rise in Gpx activity in *D. rerio* subjected to 5 mg/L of NOR for 96 h, compared to the control group [198]. Similarly, elevated NOR concentrations increased GST activity in *Carassius auratus* [199], while higher CIP concentrations increased GST activity in *C. mrigala* [196].

In terms of influencing the growth, development, and behavioral activities of organisms, Roberto et al. [172] exposed *Daphnia magna* to water containing 6.90 mg/L of ENR, LEV, and FLU for 12 days, discovering that all three types of FQs significantly inhibited the survival rate of *D. magna*. Furthermore, *D. rerio* exhibited a significant decrease in heart rate under the stress of higher concentrations of CIP and GAT [200]. Under NOR exposure (25 mg/L), it reduced the hatching rate of embryos, increased mortality and deformity rates, and interfered with the innate immune system [198]. Moreover, it has been observed that elevated concentrations of CIP not only induce decreased appetite and reduced body size in *Rhinella arenarum larvae* [201] but also lead to diminished exploratory behavior in *D. rerio* [194].

ROS remain the leading cause of DNA damage in aquatic organisms. Liu et al. [199] observed that exposure to 0.4 mg/L NOR for 7 days resulted in significant damage to the testicular DNA of male *C. auratus*; a parallel effect was noted in embryos of embryos of *D. rerio* [202]. Additionally, heightened concentrations of ENR induce a differential expression of genes related to the immune system and metabolism in the hepatopancreas of *Eriocheir sinensis* (genes for alkaline phosphatase, NF-kappa B inhibitor alpha, alpha-amylase, and beta-galactosidase-like) [203]. Furthermore, research has unveiled that FQs impact not only the replication and transcription of enzyme genes but also have the potential to induce the generation of drug-resistant bacteria and promote the production of resistant genes. These immune genes may spread through various environmental pathways, contributing to the development of multi-drug resistance in diverse organisms, highlighting an issue that deserves special attention.

### 5.3. Toxicity and Influencing Factors of FQs in Aquatic Organisms

Numerous toxicological studies have highlighted the impact of other coexisting water pollutants on the toxicity of FQs to aquatic organisms. For example, Hong et al. [189] found that the addition of microplastics (1 to 5 mg/L) in water exacerbated the toxicity of ENR to *M. verticillatum*, with a synergistic effect that correlated positively with microplastic concentration. Heavy metals, such as Pb, not only heightened the oxidative stress induced by CIP in *D. rerio* but also hindered the fish’s exploratory behavior [194]. Similarly, Jia et al. [185] observed that co-exposure to heavy metals (Cu and Cd) and FQs (ENR and CIP) exacerbated inflammation in *D. rerio* embryos. Furthermore, Zhang et al. [204] noted that variations in water pH and dissolved organic carbon (DOC) significantly affected the toxicity of CIP to *M. aeruginosa*, revealing potential differences in cell toxicity of up to 10-fold under different water conditions. These findings indicate that changes in environmental factors can significantly influence the cellular toxicity of FQs. Therefore, a comprehensive understanding of the toxic effects of FQs on aquatic organisms necessitates careful consideration of changing environmental conditions.

## 6. Conclusions

This study examined 15 different FQs in rivers, lakes, and seawater worldwide. It was found that developing countries showed markedly higher FQ residue levels than their developed counterparts, notably in CIP and NOR, reaching maximum concentrations of 542.45 μg/L and 251.14 μg/L, respectively. Researchers detected FQ bioaccumulation in aquatic organisms, including fish, crabs, oysters, shrimps, and phytoplankton across various water systems globally. Studies on the bioaccumulation patterns of FQs in organisms revealed that their physical and chemical properties (log Kow, log D, solubility, molecular weight, etc.), species differences (growth stage, gender, different tissues, etc.), and changes in water environmental factors (heavy metals, other antibiotics, dissolved organic matter, water pH, salinity, etc.) can all affect the magnitude of FQ bioaccumulation. FQs accumulating in organisms undergo biotransformation through three main metabolic pathways: ring opening, hydroxylation, and defluorination. Notably, some metabolites may exhibit higher toxicity than the parent compounds, necessitating further research into the residual concentrations and toxicity of relevant FQ metabolites.

FQs showcased varying EC_50_ values among aquatic organisms, including algae, bacteria, crustaceans, fish, mollusk, and plants. Overall, FQs exhibited higher toxicity towards bacteria, with 62.50% displaying average EC_50_ values below 10^−1^ mg/L. Their toxicity was comparatively milder towards algae and plants. Cephalopods demonstrated the least sensitivity, with EC_50_ values ranging from 31.10 to 222.60 mg/L. Among different types of FQs, LOM and ENO showed the highest toxicity, while MOX, NOR, and FLU displayed relatively weaker toxicity. The toxicity of FQs towards aquatic organisms primarily manifested in three aspects: oxidative stress, impacts on growth and development, and genetic damage. External factors such as heavy metals, microplastics, and changes in water pH could influence the toxicity of FQs towards aquatic organisms. However, research in this area is currently limited, and further investigation is warranted.

Based on the previous research findings, several key areas should be prioritized for further studies on FQs: (1) Expanding the detection range of FQs during investigations into antibiotic contamination in aquatic ecosystems is imperative. This expansion will allow for a more comprehensive assessment of the current pollution levels in these water bodies. (2) Research endeavors should encompass a broader spectrum of aquatic organisms within the food chain, unraveling the intricate processes of FQ accumulation and biomagnification. (3) Special emphasis should be placed on exploring the impacts of external environmental factors on the accumulation levels and toxicity of FQs within organisms. (4) To gain a holistic understanding of FQ toxicity towards aquatic organisms, a pivotal focus should be directed towards studying the toxicity of their metabolites.

## Figures and Tables

**Table 1:** Minimum, maximum, mean, and median concentrations of representative FQs detected in different types of surface water ^a^.

Antibiotics	Country	Place	Min (ng/L)	Max (ng/L)	Mean (ng/L)	Med (ng/L)	Frequency	References
CIP	USA	Columbia River			0.021			[9]
		Sacramento River			0.14			[9]
		Surface water			116			[28]
	……	……	……	……	……	……	……	……
	Uganda	Victoria Lake	2	41		15	91%	[44]
		Surface Water	29	88				[45]
	Vietnam	Hanoi	ND	990		ND	13%	[46]
OFL	USA	Surface Water		182				[28]
	Argentine	Surface Water			34.14			[47]
	China	Beibu Gulf	ND	0.46	0.07	0.02	58%	[48]
	……	……	……	……	……	……	……	……
	Spain	Valencia Region	1547	4778				[27]
	Sweden	Surface Water	LOQ	37.5	2.75			[49]
	Vietnam	Hanoi	ND	630		ND	38%	[46]
NOR	USA	Chesapeake Bay	59.2	94.1				[27]
	Brazil	Surface Water		285			42%	[14]
	China	Beibu Gulf	0.43	6.17	2.1	1.83	100%	[48]
	……	……	……	……	……	……	……	……
	Switzerland	Karst System		2				[42]
	Tunisia	Mediterranean Sea	ND	20,700				[27]
	Uganda	Victoria Lake	1.9	26		14	99%	[44]
ENR	USA	Chesapeake Bay	8	17				[27]
	Asia		ND	30,000	14.6			[36]
	Brazil	Doce River	73.2	566	351.27			[30]
	Croatia	Sava	4.64	80.14	21.04	6.50	100%	[50]
	……	……	……	……	……	……	……	……
		Surface Water	69.4	69.4	69.4			[51]
		Surface Water	11.8	970			89%	[52]
	Tunisia	Mediterranean Sea	4800	40,200				[27]
LOM	China	Beijing	1.1	10.9		5.2	100%	[53]
		Bohai Region	0.21	0.44	0.26	0.25	14.29%	[54]
		Dongting Lake	ND	3075	388		26.5%	[32]
	……	……	……	……	……	……	……	……
	China	Yellow River (Mainstream)	LOQ	181	91.4		51.5%	[37]
		Yellow River (Tributaries)	LOQ	212	71.8		47.6%	[37]
	France	Charmoise River	3.6	6.7	5.5			[39]
DAN	USA	Alamance County	8.31	299.62	122.83			[55]
		North Carolina	ND	1227		5.1	67%	[40]
	Brazilian	Surface Water		272			33%	[14]
	……	……	……	……	……	……	……	……
	China	Xinjinag Uygur Autonomous	0.92	4.82	2.39	2	100%	[5]
		Xiong’an New Area	ND	2.91			41%	[7]
		Yellow River (Tributaries)	LOQ	496	61.3		41.3%	[37]
PEF	China	Guangdong	2.04	3.53		2.66	88.9%	[56]
		Hong Kong River		1.51	0.56	0.52	100%	[57]
		Surface Water		323		22.56		[31]
	……	……	……	……	……	……	……	……
	China	Yangtze River (Nanjing)	ND	5.42	0.27			[58]
		Yellow River (Mainstream)	171	3144	563		63.6%	[37]
		Yellow River (Tributaries)	5.8	4467	633		66.7%	[37]
FLE	China	Dongting Lake	ND	8.88	4.79		46.5%	[32]
		Guangdong	0.89	1.43		0.94	100%	[56]
		Hong Kong River		1.07	0.51	0.52	100%	[57]
	……	……	……	……	……	……	……	……
	China	Surface Water in Basins	ND	16.7	3.5	ND		[35]
		Xinjiang Uygur Autonomous	1.1	17.15	3.77	2.2	100%	[5]
		Xiong’an New Area	ND	1.55			36%	[7]
MAR	China	Dongting Lake	ND	1.01	0.91		30%	[32]
		Guangdong	0.18	5.31		0.25	100%	[56]
		Hong Kong River		0.59	0.24	0.25	92.31%	[57]
		Liaohe River Basins	ND	40.49	5.07		24.14%	[8]
		Surface Water		16.7		0.9		[31]
		Xinjiang Uygur Autonomous	0.85	14.85	2.92	1.53	100%	[5]
	Croatia	Sava	0.54	24.53	5.75	1.16	100%	[50]
SAR	China	Dongting Lake	ND	7.94	5.51		23.5%	[32]
		Estuary		0.11				[59]
		Guangdong	ND	18.2		2.7	33.3%	[56]
	……	……	……	……	……	……	……	……
	China	Yellow River (Mainstream)	LOQ	1899	17.7		72.7%	[37]
		Yellow River (Tributaries)	LOQ	1528	20.3		73%	[37]
	Croatia	Sava	0.49	2.79	1.05	0.70	100%	[50]
	Peru	Titicaca Lake	72.7	76.5	74.2		100%	[60]
ENO	Brazilian	Surface Water	ND	386			5%	[14]
	China	Beibu Gulf	ND	2.95	1.24	0.85	94%	[48]
		Bohai Sea	ND	508	116			[39]
	……	……	……	……	……	……	……	……
	France	Charmoise River	ND	1310	134			[39]
	Malaysia	Larut River	LOQ	2.55	0.14		11.11%	[41]
	Uganda	Victoria Lake	2.9	51		25	88%	[44]
DIF	China	Beijing	ND	6.3		1.6	50%	[53]
		Changzhou	5.9	7.9		7.7	100%	[53]
		Dongting Lake	ND	4.75	2.38		45%	[32]
		Guangdong	0.84	1.24		0.85	100%	[56]
		Liaohe River Basins	ND	4.54	0.2		6.9%	[8]
		Surface Water		218.4		0.74		[31]
LEV	USA	Columbia River			1			[9]
		Sacramento River			2			[9]
	China	Chaohu Lake		89.86	<25			[61]
		Surface Water		23.4		6		[31]
	South Africa	Apies River			2.4			[62]
	Uganda	Victoria Lake	1.8	29		12	96%	[44]
MOX	USA	Sacramento River			0.012			[9]
	China	Liaohe River Basins	ND	41.1			13.79%	[8]
	China			300				[31]
	Spain		1.4	9.8			7%	[52]
FLU	China	Estuary		0.43				[59]
		Liaohe River Basins	ND	3.82	0.17		6.9%	[8]
		North South China		22.6				[42]
	……	……	……	……	……	……	……	……
	France	Surface Water		16				[63]
	Korea	Chungcheong Province		1.58				[42]
	Spain	NE Catalonia		8.9				[42]

a: The comprehensive contamination status of FQs in surface water can be found in Table S2 [5,6,7,8,9,13,14,15,16,26,27,28,29,30,31,32,33,34,35,36,37,38,39,40,41,42,43,44,45,46,47,48,49,50,51,52,53,54,55,56,57,58,59,60,61,62,63,64,65,66,67,68,69,70,71,72,73,74,75,76,77,78,79,80,81,82,83,84,85,86,87,88,89,90,91,92,93,94,95,96,97,98,99,100,101,102,103,104].

**Table 2:** The bioaccumulation of FQs in aquatic organisms from surface waters.

Antibiotics	Range (ng/g)	Mean (ng/g)	Median (ng/g)	Species	Place	References
CIP	28.51–96.22		62.37	*Halobatrachus didactulus*	Portugal (Tejo estuary)	[105]
	12.00–80.00		30.00	Bivalve	China (Taihu Lake)	[4]
	ND–30.00		9.50	Phytoplankton	China (Taihu Lake)	[4]
		176.00		Fish	Canada	[106]
	ND–112.00	37.33		*Lemna gibba*	Argentine (Luján and Moreno cities)	[47]
	3.80–4.80	4.15		*Oncorhynchus mykiss*	Peru (Lake Titicaca)	[60]
NOR	1.40–3.14	2.16		Crab	China (Beibu Gulf)	[1]
	8.70–134.00		17.00	Snail	China (Taihu Lake)	[4]
	ND–1.37			Sea cucumber	China (Dongying)	[107]
OFL	ND–0.46	0.14		Oyster	China (Beibu Gulf)	[1]
	10.63–22.50		16.57	*Dicentrarchus labrax* (adults)	Portugal (Tejo estuary)	[105]
	ND–36.00	12.00		*Lemna gibba*	Argentine (Luján and Moreno cities)	[47]
ENR	ND–0.64	0.20		Crab	China (Beibu Gulf)	[1]
	6.73–102.87	34.66	17.92	Fish	China (Guangxi)	[63]
ENO	0.09–0.24			Mitten crab	China (Dongying)	[107]
	ND–0.54	0.18		Shrimp	China (Beibu Gulf)	[1]
LOM	ND–316.51	13.04	9.99	Phytoplankton	China (Peal River)	[108]
	ND–78.66	17.52	13.53	Zooplankton	China (Peal River)	[108]
PEF	ND–1.00	0.04		Fish muscle	China (Taihu Lake)	[109]
MAR	ND–LOQ	0.01	ND	Fish muscle	China (Taihu Lake)	[109]
SAR	3.40–3.90	3.55		*Oncorhynchus mykiss*	Peru (Lake Titicaca)	[60]
	ND–0.34			*Penaeus Vannamei*	China (Dongying)	[107]

## Data Availability

Data are contained within the article and supplementary materials.

## Supplementary Materials

The following supporting information can be downloaded at: https://www.mdpi.com/article/10.3390/toxics11120966/s1, Figure S1. The structural formula of FQ; Figure S2. The metabolites and possible pathways of GAT (a), MOX (b), LEV (c), CIP (d), and ENR (e) biodegradation in aquatic organisms. Table S1: Physicochemical properties of selected FQs [5,6,7,8,9]; Table S2. Minimum, maximum, mean, and median concentrations of representative FQs detected in different surface water [5,6,7,8,9,13,14,15,16,26,27,28,29,30,31,32,33,34,35,36,37,38,39,40,41,42,43,44,45,46,47,48,49,50,51,52,53,54,55,56,57,58,59,60,61,62,63,64,65,66,67,68,69,70,71,72,73,74,75,76,77,78,79,80,81,82,83,84,85,86,87,88,89,90,91,92,93,94,95,96,97,98,99,100,101,102,103,104]; Table S3. Toxicity of selected fluoroquinolone towards various trophic groups of organisms [19,28,120,148,149,150,151,152,153,154,155,156,157,158,159,160,161,162,163,164,165,166,167,168,169,170,171,172,173,174,175,176,177,178,179,180,181].
