# Supplementary material for: Occurrence, Bioaccumulation, Metabolism and Ecotoxicity of Fluoroquinolones in the Aquatic Environment: A Review
Source: Toxics. 2023 Nov 29;11(12):966. doi: 10.3390/toxics11120966 (PMC10747319; doi:10.3390/toxics11120966)
Supplement: Supplementary file 1 [file toxics-11-00966-s001.zip › toxics-2730550-supplementary.pdf]

toxics

Supplementary Materials: Occurrence, bioaccumulation, metabolism and ecotoxicity of fluoroquinolones in the aquatic environment: A review

Mengnan Shen 1, Yi Hu 1, Ke Zhao 1, Chenyang Li 1, Binshuo Liu 1, Ming Li 1, Chen Lyu 1, Lei Sun 2,* and Shuang Zhong 3,*

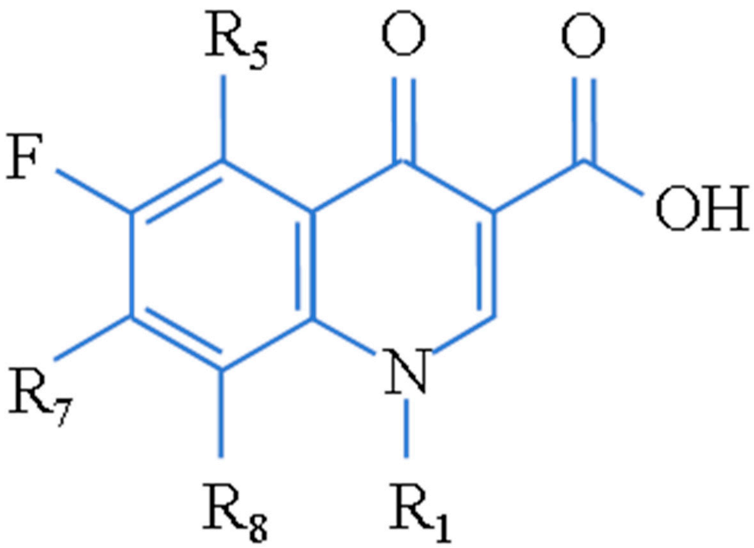

Figure S1. The structural formula of FQ.

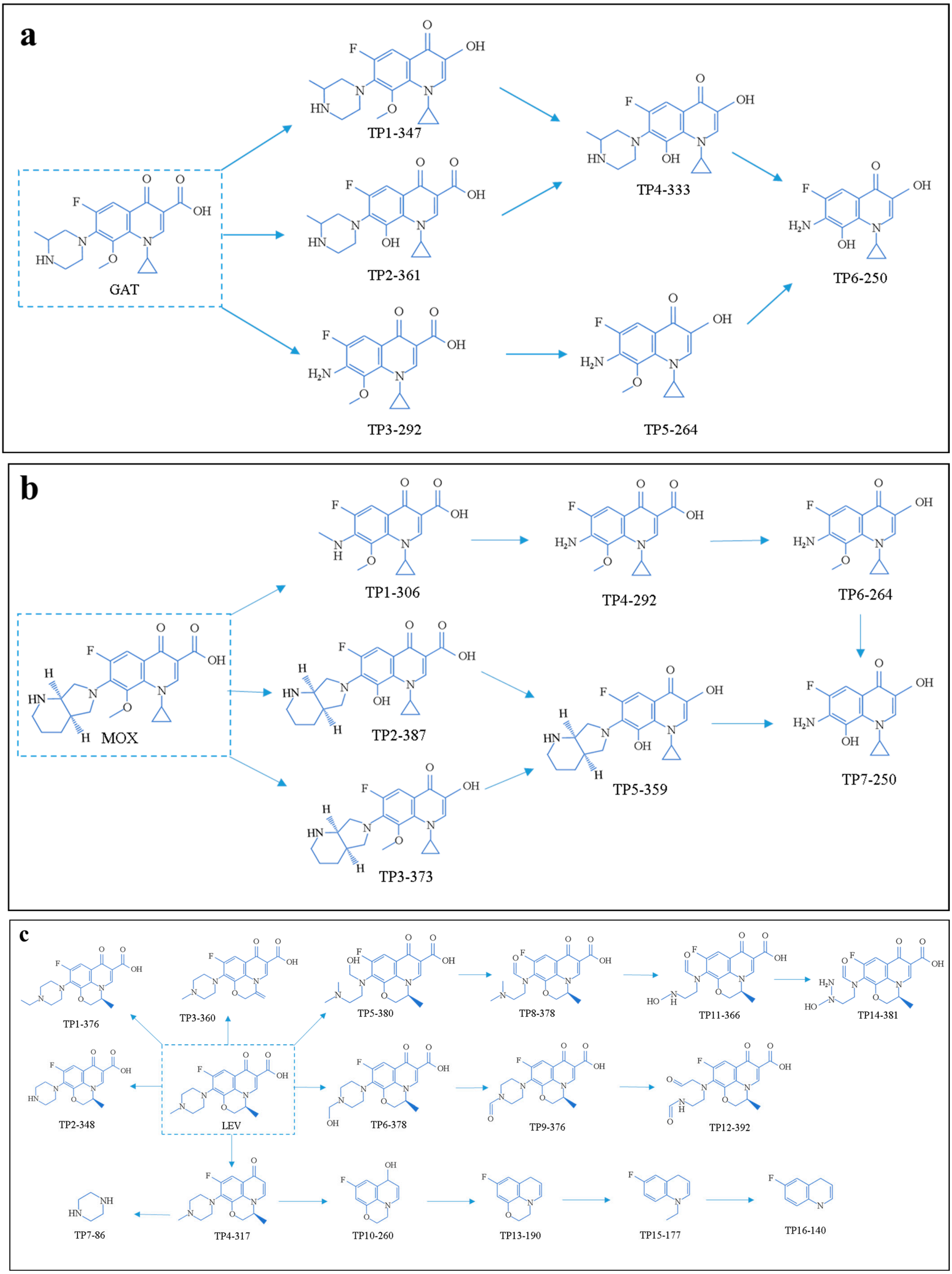
a
GAT
TP1-347
TP2-361
TP3-292
TP4-333
TP5-264
TP6-250
b
MOX
TP1-306
TP2-387
TP3-373
TP4-292
TP5-359
TP6-264
TP7-250
c
LEV
TP1-376
TP2-348
TP3-360
TP4-317
TP5-380
TP6-378
TP7-86
TP8-378
TP9-376
TP10-260
TP11-366
TP12-392
TP13-190
TP14-381
TP15-177
TP16-140

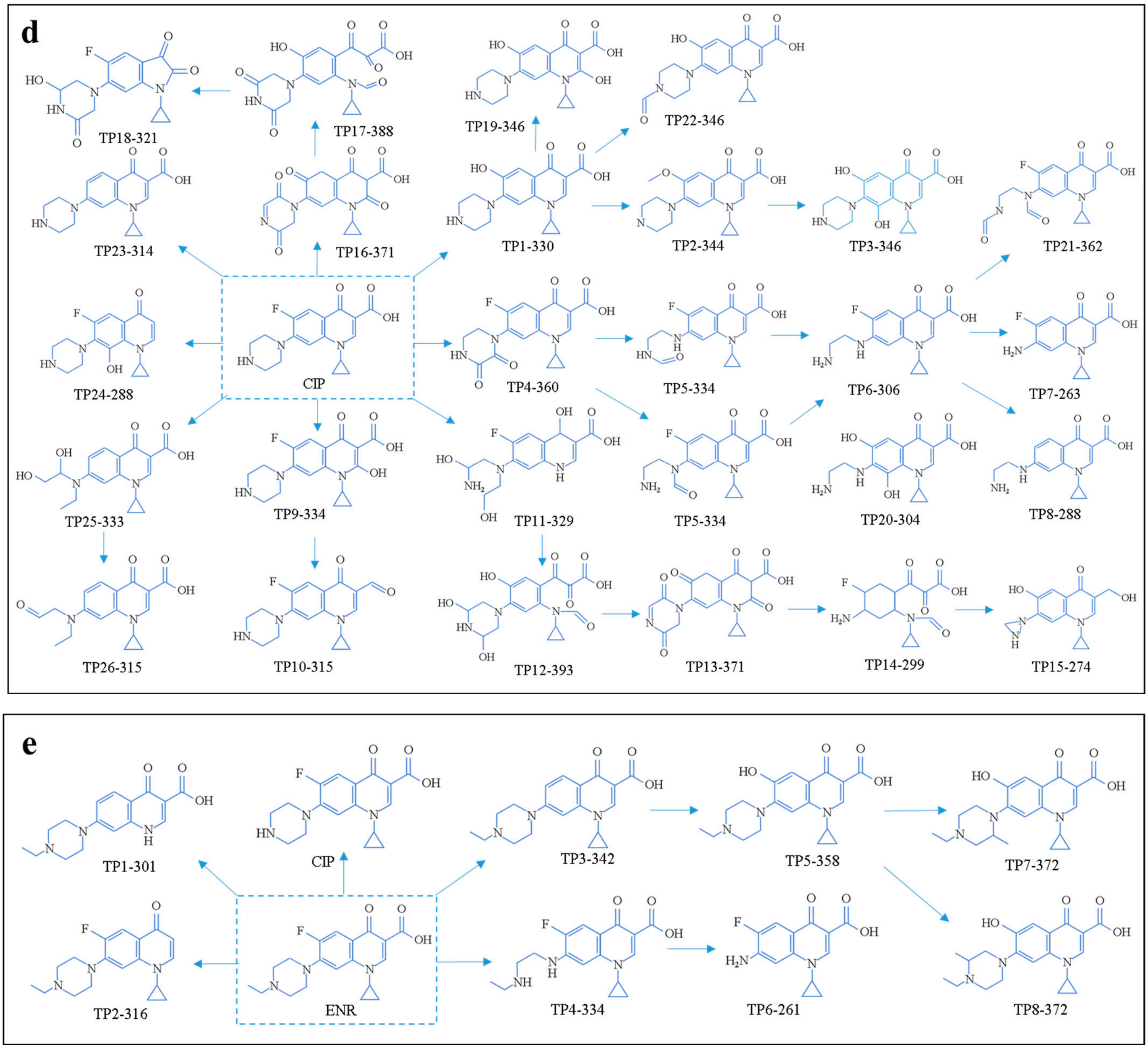

Figure S2. The metabolites and possible pathways of GAT (a), MOX (b), LEV (c), CIP (d), and ENR (e) biodegradation in aquatic organisms.Table S1. Physicochemical properties of selected FQs.

Table S2. Minimum, maximum, mean, and median concentrations of representative FQs detected in different surface water.

Compound	Molecular Formula	Chemical Structure	CAS number	Molecular Mass	Log Kow	pKa	References

Ciprofloxacin	$C_{17}H_{18}FN_3O_3$	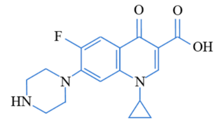	85721-33-	331.34	0.28	5.56/8.77	[1]
			1				
Ofloxacin	C18H20FN3O4	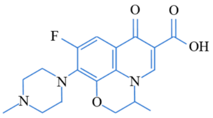	82419-36-	361.38	-0.39	5.35/6.72	[1]
			1-0				
Norfloxacin	$C_{16}H_{18}FN_3O_3$	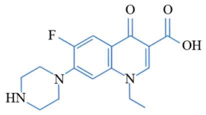	70458-96-	319.33	-1.03	5.58/8.77	[1]
			7				
Enrofloxacin	$C_{19}H_{22}FN_3O_3$	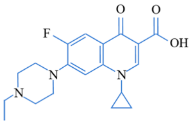	93106-60-	359.39	1.1	6.19/7.59	[2]
			6				
Lomefloxacin	$C_{17}H_{19}F_2N_3O_3$	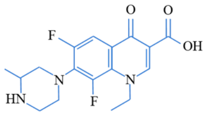	98079-51-	351.35	-0.3	-	[2]
			7				
Danofloxacin	$C_{19}H_{20}FN_3O_3$	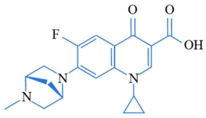	112398-	357.38	0.44	5.65/6.73	[1]
			08-0				
Pefloxacin	C17H20FN3O3	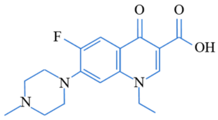	70458-92-	333.36	0.27	5.55/7.01	[1]
			3				

Compound	Molecular Formula	Chemical Structure	CAS number	Molecular Mass	Log Kow	pKa	References
Fleroxacin	$C_{17}H_{18}F_3N_3O_3$	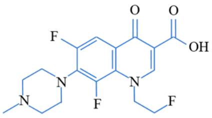	79660-72-3	369.34	0.24	5.44/6.06	[1]
Marbofloxacin	$C_{17}H_{19}FN_4O_4$	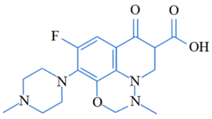	115550-35-1	362.35	-2.92	5.38/6.16	[1]
Sarafloxacin	$C_{20}H_{17}F_2N_3O_3$	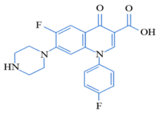	98105-99-8	385.36	1.07	5.55/8.76	[1]
Enoxacin	$C_{15}H_{17}FN_4O_3$	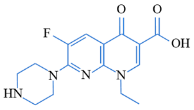	74011-58-8	320.32	-0.20	5.50/8.59	[3]
Difloxacin	$C_{21}H_{19}F_2N_3O_3$	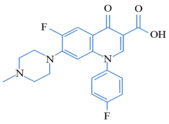	98106-17-3	399.39	0.89	-	[4]
Levofloxacin	$C_{18}H_{20}FN_3O_4$	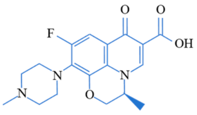	100986-85-4	361.37	0.51	5.7	[5]

Moxifloxacin	$C_{21}H_{24}FN_3O_4$	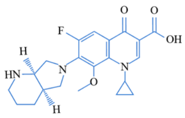	151096-09-2	401.43	0.95	-	[4]
Flumequine	$C_{14}H_{12}FNO_3$	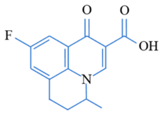	42835-25-6	261.25	1.60	6.50	[3]

Antibiotics	Country	Place	Min (ng/L)	Max (ng/L)	Mean (ng/L)	Med (ng/L)	Frequency	References
Ciprofloxacin	USA	Columbia River			0.021			[5]
		Sacramento River			0.14			[5]
		Surface water			116			[6]
	Australia			1300		70	30	[6]
	Argentine				102.04			[7]
	Bangladesh	Aftabnagar Lake	ND	88	30		67	[8]
		Rampura Canal	455	1407	766		100	[8]
		Trimohoni pump	ND	16	0		67	[8]
		Trimohoni River	466	754	624		100	[8]
	Brazil	Doce river watershed	171.93	3993.61	2277.61			[9]
	China	Beibu Gulf	ND	2.76	0.57	0.46	71	[10]
		Beijing		30.7	<10			[11]
		Beijing	ND	12.8		3.5	63	[12]
		Beiyangdian Lake	19.02	32.03	27.75	29.77	100	[13]
		Bohai Estuarine water	17	200	75	48		[14]
		Bohai region	0.42	21.56	2.27	1.48	54.29	[15]
		Bohai Sea	ND	346	101			[16]
		Bohai Seawater	5.2	85	38	42		[14]
		Chaohu Lake		23.15	<2.5			[17]
		Chaohu Lake	ND	21.35	3.47	ND	16	[18]
		Chaohu Lake			23.2			[19]
		Changzhou	1.5	4.7		4		[12]
		Dongting Lake	ND	27.02	0.64		2.37	[20]

Continuation S2

Antibiotics	Country	Place	Min (ng/L)	Max (ng/L)	Mean (ng/L)	Med (ng/L)	Frequency	References
		Dongting Lake	ND	134	29.9		70%	[21]
		Eastern China	0.019	30	5.56	1.2	100%	[22]
		Estuary		2.28				[23]
		Guangdong	ND	43.76		1.1	77.8%	[24]
		Guangxi	3	<7	<4			[25]
		Guilin	142.59	244.63	177.21	171.15	100%	[26]
		Haikou	0.12	0.63				[27]
		Hanjiang River	ND	4.4				[19]
		Hetao Irrigation District	0.83	161.47	14.42		100%	[28]
		Hong Kong River		16.74	1.55	1.2	100%	[29]
		Houguanxia Lake			6.3			[30]
		Huangpu River	ND	34.2				[19]
		Jianghan Plain	4.46	96	25.14			[16]
		Laizhou Bay			66			[31]
		Liaohe River Basin	ND	5.02	0.17		3.45%	[4]
		Mediterranean River	21.3	59.16				[19]
		Nansi Lake	ND	0.85	0.1			[32]
		North China Plain	ND	10.71	0.19		2.47%	[33]
		Peal River		365				[34]
		Peal River	4.24	49.9	9.94		100%	[35]
		Shatian Lake	1.2	7.6	2.72	2.4	100%	[36]
		Songhua River		80.54				[34]
		Surface water	0.1	2850	153.5	26.7		[37]

Continuation S2

Antibiotics	Country	Place	Min (ng/L)	Max (ng/L)	Mean (ng/L)	Med (ng/L)	Frequency	References
		Surface Water		1729		11.44		[38]
		Surface Water		551.4	68.99			[6]
		Surface Water	ND	450.2	145.13	134.2	83.33%	[39]
		Surface water in basins	0.1	230	57.96	15.3		[37]
		Suzhou	ND	90.5	10.02	4.73	96.09%	[40]
		Tianjin	0.35	14.4	1.97	1.29	100%	[41]
		Wangyang River	238.3	551.4				[42]
		WWPTs		＜100				[43]
		Xiaoqing River	ND	552.38	18.16			[44]
		Xiaoqing River	ND	877	125		82.1%	[45]
		Xiaoqing River (Mainstream)	LOQ	2715	95.7		59.7%	[46]
		Xiaoqing River (Tributaries)	LOQ	1857	83.7		56.9%	[46]
		Xinjiang Uygur Autonomous	2.1	3.35	2.72		100%	[1]
		Xiong'an New Area	ND	15.77			85%	[3]
		Yang River		1.20				[23]
		Yangjie River	0.77	4.32				[19]
		Yangtze River	ND	0.94	0.34			[16]
		Yangtze River		283.5				[34]
		Yangtze River	ND	46.68	8.1			[47]
		Yangtze River	ND	0.94	0.34		67%	[48]
		Yellow River			37.73			[34]
		Yellow River (Mainstream)	LOQ	1476	406		36.4%	[46]
		Yellow River (Tributaries)	LOQ	4467	633		46%	[46]
		Yellow River (Henan)	ND	10.1	3.03		66.67%	[49]

Continuation S2

Antibiotics	Country	Place	Min (ng/L)	Max (ng/L)	Mean (ng/L)	Med (ng/L)	Frequency	References
		Zhejiang	0.1	<20	<10			[50]
	Costa Rica	Surface Water	160	1330	160		18%	[42]
	Croatia	Sava	0.39	5.38	2.04	1.24	100%	[51]
	Cyprus	Surface Water	252.3	316.8	284.55			[52]
	Finland	Surface Water	38.4	43.2	40.8			[52]
		Surface Water			20			[31]
	France	Charmoise River	5	1523	288			[16]
		Surface Water		1523	90.42			[6]
		Surface Water		25				[53]
	Germany	Surface Water	43.8	230.6	137.2			[52]
	Global	Surface Water	ND	542452				[54]
	India	Chennai city	10.5	186.1	31.95		100%	[55]
		Ganges	LOQ	26.8	7.7	5	63.3%	[56]
	Iran	Ekbatan	127	248				[31]
		Firozaba River			656.8			[19]
		Kan River			22.7			[19, 31]
		Kan River	552.6	796.2				[31]
	Ireland	Surface Water	234	259.8	246.9			[52]
	Italy	Surface Water			7.7			[57]
		Surface Water		16	8.8			[6]
	Kenya	Jujia drain	20300	75700	48700	52300	100%	[58]
		Nzoia River Basin		8.6	0.9		23.81%	[59]
		Surface Water	12	510				[19]
		Surface Water	500	2800	1775		100%	[60]

Continuation S2

Antibiotics	Country	Place	Min (ng/L)	Max (ng/L)	Mean (ng/L)	Med (ng/L)	Frequency	References
		Surface Water	ND	1300				[16]
	Korea	Chungcheong Province		0.52				[53]
	Malaysia	Gombak River	225.18	299.88	267.2			[61]
		Larut River	LOQ	577.97	56.41		55.56%	[62]
		Lui River	52.5	138.7				[63]
	Norway	Surface Water	159.2	159.2	159.2			[52]
	Pakistan	Lahore		0.07				[53]
	Peru	Titicaca Lake	85.5	408.2	201		100%	[64]
	Poland	Surface Water	12	182	99.25		100%	[65]
		Surface Water		277	24.83			[6]
	Portugal	Leca River	ND	339				[16]
		Surface Water		88.7		88.7	1.82%	[6]
		Tejo Estuary	1.56	7.14				[19]
		Surface Water			88.7			[57]
		Surface Water	231.4	584.9	424.53			[52]
	Romania	Cluj Napoca		77.53				[53]
		Surface Water			15			[57]
	Spain	Baix Fluvià			211.8			[31]
		Barcelona		443				[53]
		Ebro delta	80	5875	2027.5			[66]
		Mijares River	ND	1105				[67]
		NE Catalonia		77.2				[53]
		Surface Water		740	30.99			[6]
		Ter River	48.2	72.4				[19]

Continuation S2

Antibiotics	Country	Place	Min (ng/L)	Max (ng/L)	Mean (ng/L)	Med (ng/L)	Frequency	References
		Surface Water	1	93.9				[57]
		Surface Water	200.3	200.3				[52]
		Surface Water	LOQ	33.66			29%	[68]
	Sweden	Surface Water			17.78			[57]
		Surface Water	LOQ	560	48.64	5.05		[69]
	Turkey	Surface Water		13567	50.55			[6]
	Uganda	Victoria Lake	2	41		15	91%	[70]
		Surface Water	29	88				[71]
	Vietnam	Hanoi	ND	990		ND	13%	[72]
Ofloxacin	USA	Surface Water		182				[6]
	Argentine	Surface Water			34.14			[7]
	China	Beibu Gulf	ND	0.46	0.07	0.02	58%	[10]
		Beijing		717.3		60.9		[11]
		Beijing	2	200.6		4.2	100%	[12]
		Beiyangdian Lake	11	11.45	11.27	11.28	100%	[13]
		Bohai Estuarine	3.2	30	9.3	9.2		[14]
		Bohai region	0.21	43.63	1.36	0.38	3.97%	[15]
		Bohai Sea (North China)	ND	45.4	9.9			[16]
		Bohai Seawater	3	11	6.6	6.4		[14]
		Changzhou	3.6	8.2		3.1	100%	[12]
		Chaohu Lake			383.4			[31]
		Chaohu Lake	ND	50.6				[19]
		Dianchi Lake	ND	713.6				[31]
		Dongting Lake	6.77	943.49	87.15		100%	[20]

Continuation S2

Antibiotics	Country	Place	Min (ng/L)	Max (ng/L)	Mean (ng/L)	Med (ng/L)	Frequency	References
		Dongting Lake	ND	LOQ	LOQ		20%	[21]
		Estuary		2.63				[23]
		Fenhe River	18.21	283.34	＜106.74			[73]
		Fuxian Lake	0.77	7.3	3.4		100%	[74]
		Guangdong	0.57	17.31		2.58	100%	[24]
		Guangxi	50	＜650	＜150			[25]
		Guilin	ND	290.78	96.07	ND	40%	[26]
		Hanjiang River	ND	9.5				[19]
		Hetao Irrigation District	0.82	253.51	12.92		100%	[28]
		Hong Kong River		12.98	1.49	1.14	100%	[29]
		Huangpu River	ND	28.5	6.5			[16]
		Huangpu River	ND	28.5				[19]
		Hubei Province	2.21	127.4				[31]
		Jianghan Plain	ND	45.3	9.03			[16]
		Jiaozhou Bay			16.7			[75]
		Kaidu River and Boaten	ND	＜5			80%	[76]
		Laizhou Bay	ND	6.2				[19]
		Liaohe River Basin	ND	27.21	3.68		27.59%	[4]
		Liaohe River (Jilin)			67.1			[31]
		Main River	ND	4.3				[19]
		Nansi Lake	ND	50	3.13		85.33%	[32]
		Pearl River	0.6	60.3	8.52		100%	[35]
		Pearl River			6.93			[31]
		Pearl River	53	108				[19]

Continuation S2

Antibiotics	Country	Place	Min (ng/L)	Max (ng/L)	Mean (ng/L)	Med (ng/L)	Frequency	References
		Shatian Lake	0.1	0.6	0.25	0.2	100%	[36]
		Songhua River		14.1				[34]
		Surface Water	0.1	5100	134.8	15.2		[37]
		Surface Water		127400		11.02		[38]
		Surface Water		11734.6				[6]
		Surface Water	ND	5.98	3.18	2.62	71.4%	[77]
		Surface Water in Basins	ND	118.1	22.67	6.4		[37]
		Suzhou	ND	195	10.18	3.47	99.22%	[40]
		Taihu Lake	＜1	＜1	＜1			[78]
		Taihu Lake	7.45	17.01				[31]
		Tianjin	LOQ	44.4	3.3	1.44	100%	[41]
		Wanfeng Lake		246.96	169.48	149.58	100%	[79]
		Wangyang River	668.3	11734.6				[80]
		Wuhan City	LOQ	4.4	1.45		66.67%	[30]
		WWTPs		＜7500				[43]
		Xiaoqing River	ND	6374.45	241.39			[44]
		Xiaoqing River	0.5	240	38.2		100%	[45]
		Xiaoqing River (Mainstream)	4.48	368	64.9		67.7%	[46]
		Xiaoqing Rive (Tributaries)	3.6	1371	54.1		60%	[46]
		Xinjiang Uygur Autonomous	0.75	3.55	1.39	1.17	100%	[1]
		Xiong'an New Area	ND	76.25			69%	[3]
		Yang River		12.95				[23]
		Yangtze River	ND	0.82	0.32		75%	[48]
		Yangtze River	ND	48.92	7.89			[47]

Continuation S2

Antibiotics	Country	Place	Min (ng/L)	Max (ng/L)	Mean (ng/L)	Med (ng/L)	Frequency	References
		Yangtze River		30.6				[34]
		Yangtze River	ND	0.82	0.32			[16]
		Yangtze River (Nanjing)	ND	34.66	3.27			[81]
		Yanjie River	0.14	4.49				[80]
		Yellow River			119			[31]
		Yellow River (Mainstream)	3.2	329	10.9		54.5%	[46]
		Yellow River (Tributaries)	LOQ	491	11.4		57.1%	[46]
		Yellow River (Henan)	ND	511	11.65		72%	[49]
		Zhejiang	0.1	97.5	<3			[50]
		Zhuhai			114.9			[82]
	Costa Rica	Surface Water	90	1250	670		7%	[42]
	Cyprus	Surface Water	196.7	305.1	250.9			[52]
	Finland	Surface Water	20	22.8	21.4			[52]
	France	Charmoise River	100	2888	1209			[16]
		Surface Water		19.7				[53]
		Surface Water		2888	113.52			[6]
	Germany	Surface Water	ND	66.5	33.25			[52]
	Global	Surface Water	ND	6840				[54]
	India	Chennai City	0.8	222.8	80.38		100%	[55]
		Ganges	LOQ	1.4	1	1.1	11.1%	[56]
		Kshipra River	640	1460	985.71			[83]
		Surface Water		11000	4284.55			[6]
	Ireland	Surface Water	39.9	65.4	52.65			[52]

Continuation S2

Antibiotics	Country	Place	Min (ng/L)	Max (ng/L)	Mean (ng/L)	Med (ng/L)	Frequency	References
	Italy	Surface Water		18.06	10.09			[6]
	Korea	Chungcheong Province		0.70				[53]
	Malaysia	Larut River	LOQ	253.09	21.47		33.33%	[62]
	Norway	Surface Water	27.1	27.1	27.1			[52]
	Poland	Bialka River	0.73	13.71	3.47	2.16		[84]
		Surface Water		89	8.83			[6]
		River	4	32	18.85		100%	[65]
	Portugal	Leca River	ND	120				[16]
		Surface Water	89.7	184.9	135.8			[52]
	Spain	Barcelona		79.5				[53]
		Ebro Delta	137	1161	535			[66]
		Ebro River	ND	78.6				[80]
		NE Catalonia		18.2				[53]
		Surface Water		8770	210.74			[6]
		Surface Water	142.3	142.3	142.3			[52]
		Ter River	100.7	137.6				[80]
		Tributaries	ND	79.9				[80]
		Valencia Region	1547	4778				[80]
	Sweden	Surface Water	LOQ	37.5	2.75			[69]
	United States	Surface Water		13.53				[53]
	Vietnam	Hanoi	ND	630		ND	38%	[72]
Norfloxacin	USA	Chesapeake Bay	59.2	94.1				[80]
	Brazil	Surface Water		285			42%	[85]
	China	Beibu Gulf	0.43	6.17	2.1	1.83	100%	[10]

Continuation S2

Antibiotics	Country	Place	Min (ng/L)	Max (ng/L)	Mean (ng/L)	Med (ng/L)	Frequency	References
		Beijing		290.2	<50			[11]
		Beijing	ND	47		28.5	88%	[12]
		Beiyangdian Lake	16.9	22.95	19.14	18.4	100%	[13]
		Bohai Estuarine	40	230	146	137		[14]
		Bohai Region	0.33	25.52	1.65	0.92	68.57%	[15]
		Bohai (North China)	ND	572	118			[16]
		Bohai Seawater	8.4	134	57	59		[14]
		Chaohu Lake		24.18	<5			[17]
		Changzhou	63.8	70.9		69.3	100%	[12]
		Chaohu Lake	ND	19.11	1.59	ND	8%	[18]
		Chaohu Lake	ND	70.2				[80]
		Dongting Lake	ND	6.22	4.52		40%	[21]
		Dongting Lake	ND	12.17	0.29		2.37%	[20]
		Eastern China	0.07	53	8.93	0.75	100%	[22]
		Guangdong	1.77	53.81		4.65	88.9%	[24]
		Guangxi	3	<7	<4			[25]
		Guilin	ND	187.56	110.17	177.61	60%	[26]
		Hangzhou	7	12.9				[27]
		Hanjiang River	ND	6.4				[80]
		Hetao Irrigation District	6.51	220.91	43.71		100%	[28]
		Hong Kong River		7.38	1.13	0.97	100%	[29]
		Huangpu River	ND	0.2				[80]
		Jianghan Plain	9.57	277	65.87			[16]
		Laizhou Bay	7.5	103				[80]

Continuation S2

Antibiotics	Country	Place	Min (ng/L)	Max (ng/L)	Mean (ng/L)	Med (ng/L)	Frequency	References
		Liaohe River	ND	13.65	0.65		6.9%	[4]
		Nanjing	146.72	290.2				[31]
		Nansi Lake	ND	1	0.24		52.33%	[32]
		North China	ND	7.92	1.96		51.85%	[33]
		Pearl River			68.06			[31]
		Pearl River	5.49	27.2	10.33		100%	[35]
		Shatian Lake	1.2	7.4	2.71	2.2	100%	[36]
		Songhua River		268.86				[34]
		Surface Water		6800		10.36		[38]
		Surface Water	0.1	6800	192.8	37.1		[37]
		Surface Water in Basins	1.8	64.4	29.64	22.9		[37]
		Suzhou	1.17	556	100.5	57.59	100%	[40]
		Taihu Lake	15.83	56.22				[31]
		Taihu Lake	<1	<1	<1			[78]
		Tianjin	1.42	16.6	5.76	5.1	100%	[41]
		Wangyang River	199.4	617.1				[80]
		WWTPs		<2500				[43]
		Xiaoqing River	ND	888.07	31			[44]
		Xiaoqing River (Mainstream)	LOQ	2477	13.9		35.5%	[46]
		Xiaoqing River (Tributaries)	LOQ	9173	11.1		33.8%	[46]
		Xinjiang Uygur Autonomous	2.91	9.85	4.81	4.33	100%	[1]
		Xiong'an New Area	ND	30.66			67%	[3]
		Yangtze River	0.11	2.37				[80]
		Yangtze River	ND	0.83	0.32		67%	[48]

Continuation S2

Antibiotics	Country	Place	Min (ng/L)	Max (ng/L)	Mean (ng/L)	Med (ng/L)	Frequency	References
		Yangtze River		278.2				[34]
		Yangtze River (Nanjing)	146.72	290.2	180.93		100%	[81]
		Yellow River			26.03			[34]
		Yellow River	6.14	382.3	43.47	23.84	100%	[2]
		Yellow River			327			[31]
		Yellow River (Tributaries)	LOQ	10088	524		33.3%	[46]
		Yellow River (Henan)	ND	3450	86.13		96%	[49]
		Zhejiang	0.1	＜10	＜2			[50]
	France	Charmoise River	14	1261	289			[16]
		Seine River			163			[31]
	Global	Surface Water	ND	251137				[54]
	India	Chennai City	0.5	20.9	4.33		88%	[55]
		Kshipra River	640	980	752.86			[83]
	Kenya	Juja Drain	10500	52600	37500	41400	93%	[58]
		Surface Water	600	4900	2325		100%	[60]
		Surface Water	ND	2200				[16]
	Korea	Chungcheong Province		1.27				[53]
	Malaysia	Larut River	LOQ	2.55	0.37		33.33%	[62]
	Poland	River	LOQ	95	23.75		25%	[65]
	Romania	Cluj Napoca		8.15				[53]
	Spain	Barcelona		123				[53]
		Ebro Delta	25	497	236			[66]
		Mijares River	ND	940				[67]
		NE Catalonia		30.3				[53]

Continuation S2

Antibiotics	Country	Place	Min (ng/L)	Max (ng/L)	Mean (ng/L)	Med (ng/L)	Frequency	References
		Surface Water	LOQ	3.2			4%	[68]
	Switzerland	Karst System		2				[53]
	Tunisia	Mediterranean Sea	ND	20700				[80]
	Uganda	Victoria Lake	1.9	26		14	99%	[70]
Enrofloxacin	USA	Chesapeake Bay	8	17				[80]
	Asia		ND	30000	14.6			[86]
	Brazil	Doce River Watershed	73.2	566	351.27			[9]
		Surface Water		71			50%	[85]
	China	Beibu Gulf	ND	1.1	0.25	0.19	87%	[10]
		Beijing	4.8	29.9		18.3	100%	[12]
		Bohai Estuarine	3.9	27	11	9		[14]
		Bohai Region	0.21	16.79	1	0.61	69.29%	[15]
		Bohai Sea (North China)	ND	24.6	10.6			[16]
		Bohai Seawater	3	11	6.2	6.1		[14]
		Chaohu Lake		25.16	<6			[17]
		Chaohu Lake	ND	4.7				[80]
		Changzhou	20.2	26.8		22.8	100%	[12]
		Dongting Lake	ND	4.82	3.61		23.5%	[21]
		Dongting Lake	ND	63.75	2.7		16.67%	[20]
		Eastern China	0.02	24.1	3.59	0.65	100%	[22]
		Estuary		0.14				[23]
		Guangdong	0.93	9.1		1.25	100%	[24]
		Guangxi	3	<7	<4			[25]
		Guilin	184.71	673.13	342.66	248.27	100%	[26]

Continuation S2

Antibiotics	Country	Place	Min (ng/L)	Max (ng/L)	Mean (ng/L)	Med (ng/L)	Frequency	References
		Hangzhou	10.5	18.7				[27]
		Hanjiang River	ND	3.6				[80]
		Hetao Irrigation District	0.81	14.24	4.64		100%	[28]
		Hong Kong River		3.85	0.54	0.49	1005	[29]
		Honghu Lake		＜5				[87]
		Huangpu River	ND	14.6	2.8			[16]
		Jianghan Plain	ND	136	35.56			[16]
		Laizhou Bay	ND	7.6				[80]
		Laizhou Bay			209			[31]
		Liaohe River Basin	ND	5.47	0.35		13.79%	[4]
		Mediterranean River	2.87	154.34				[80]
		Nansi Lake	ND	0.94	0.21		53.33%	[32]
		North China	ND	33.29	1.43		45.68%	[33]
		Shatian Lake	1.1	15	6.05	4.9	100%	[36]
		Songhua River		16.4				[34]
		Surface Water		5681.9		9.18		[38]
		Surface Water	ND	900	36.8	6.6		[37]
		Surface Water in Basins	1.4	59.7	16.63	8.2		[37]
		Suzhou	0.68	67.3	14.76	11.19	100%	[40]
		Taihu Lake	＜1	＜1	＜1			[78]
		Wangyang River	242.6	978.8				[80]
		Xiaoqing River	ND	146.51	3.63			[44]
		Xiaoqing River (Mainstream)	LOQ	11890	72.1		72.1%	[46]
		Xiaoqing River (Tributaries)	LOQ	15375	75.4		60%	[46]

Continuation S2

Antibiotics	Country	Place	Min (ng/L)	Max (ng/L)	Mean (ng/L)	Med (ng/L)	Frequency	References
		Xiong'an New Area	ND	2.82			51%	[3]
		Yang River		0.61				[23]
		Yangjie River	0.53	5.56				[80]
		Yangtze River	ND	0.89	0.4		83%	[48]
		Yangtze River (Nanjing)	3	26.24	11.92		100%	[81]
		Yellow River	8.83	206.7	27.35	18.63	100%	[2]
		Yellow River (Mainstream)	LOQ	13462	74.7		63.6%	[46]
		Yellow River (Tributaries)	LOQ	11900	297		63.5%	[46]
	Croatia	Sava	4.64	80.14	21.04	6.50	100%	[51]
	France	Surface Water		9				[53]
	Global	Surface Water	ND	181609				[54]
	India	Chennai City	0.5	4.9	1.53		96%	[55]
	Korea	Chungcheong Province		0.19				[53]
	Malaysia	Larut River	LOQ	34.3	3.63		44.44%	[62]
	Peru	Titicaca Lake	56.2	63	60.7		100%	[64]
	South Africa	Apies River			3.12			[88]
	Spain	Barcelona		264				[53]
		NE Catalonia		60.1				[53]
		Surface Water	69.4	69.4	69.4			[52]
		Surface Water	11.8	970			89%	[68]
	Tunisia	Mediterranean Sea	4800	40200				[80]
Lomefloxacin	China	Beijing	1.1	10.9		5.2	100%	[12]
		Bohai Region	0.21	0.44	0.26	0.25	14.29%	[15]
		Dongting Lake	ND	3075	388		26.5%	[21]

Continuation S2

Antibiotics	Country	Place	Min (ng/L)	Max (ng/L)	Mean (ng/L)	Med (ng/L)	Frequency	References
		Estuary		1				[23]
		Guangdong	0.85	2.61		0.89	100%	[24]
		Hetao Irrigation District	LOQ	1.37	0.18		56.5%	[28]
		Hong Kong River		1.77	0.62	0.43	100%	[29]
		Jianghan Plain	ND	36.4	9.98			[16]
		Liaohe River Basin	ND	14.61	1.15		20.69%	[4]
		Surface Water		1170.4		5.63		[38]
		Surface Water	ND	1735.4	37.3	3.8		[37]
		Surface Water in Basins	ND	5.8	1.29	ND		[37]
		Xiaoqing River (Mainstream)	LOQ	214	89.1		41.9%	[46]
		Xiaoqing River (Tributaries)	LOQ	233	76.3		43.1%	[46]
		Yang River		2.37				[23]
		Yellow River	ND	1.94	0.29	0.2	88%	[2]
		Yellow River (Mainstream)	LOQ	181	91.4		51.5%	[46]
		Yellow River (Tributaries)	LOQ	212	71.8		47.6%	[46]
	France	Charmoise River	3.6	6.7	5.5			[16]
Danofloxacin	USA	Alamance County	8.31	299.62	122.83			[89]
		North Carolina	ND	1227		5.1	67%	[90]
	Brazilian	Surface Water		272			33%	[85]
	China	Guangdong	1.6	189.53		1.97	100%	[24]
		Liaohe River Basins	ND	32.38	2.36		27.59%	[4]
		Surface Water		603.29		62.83		[38]
		Xiaoqing River (Mainstream)	LOQ	4111	50.6		48.4%	[46]
		Xiaoqing River (Tributaries)	LOQ	231	61.5		46.2%	[46]

Continuation S2

Antibiotics	Country	Place	Min (ng/L)	Max (ng/L)	Mean (ng/L)	Med (ng/L)	Frequency	References
		Xinjinag Uygur Autonomous	0.92	4.82	2.39	2	100%	[1]
		Xiong'an New Area	ND	2.91			41%	[3]
		Yellow River (Tributaries)	LOQ	496	61.3		41.3%	[46]
Pefloxacin	China	Guangdong	2.04	3.53		2.66	88.9%	[24]
		Hong Kong River		1.51	0.56	0.52	100%	[29]
		Surface Water		323		22.56		[38]
		Xiaoqing River (Mainstream)	7.6	4154	903		72.6%	[46]
		Xiaoqing River (Tributaries)	5.3	4870	886		69.2%	[46]
		Xinjiang Uygur Autonomous	2.34	17.6	8.97	6.3	100%	[1]
		Yangtze River (Nanjing)	ND	5.42	0.27			[81]
		Yellow River (Mainstream)	171	3144	563		63.6%	[46]
		Yellow River (Tributaries)	5.8	4467	633		66.7%	[46]
Fleroxacin	China	Dongting Lake	ND	8.88	4.79		46.5%	[21]
		Guangdong	0.89	1.43		0.94	100%	[24]
		Hong Kong River		1.07	0.51	0.52	100%	[29]
		Liaohe River Basins	ND	3.51	0.64		20.69%	[4]
		Shatian Lake	0.4	0.6	0.45	0.4	100%	[36]
		Surface Water		252		3.3		[38]
		Surface Water	ND	309.4	32.2	7.3		[37]
		Surface Water in Basins	ND	16.7	3.5	ND		[37]
		Xinjiang Uygur Autonomous	1.1	17.15	3.77	2.2	100%	[1]
		Xiong'an New Area	ND	1.55			36%	[3]
Marbofloxacin	China	Dongting Lake	ND	1.01	0.91		30%	[21]
		Guangdong	0.18	5.31		0.25	100%	[24]

Continuation S2

Antibiotics	Country	Place	Min (ng/L)	Max (ng/L)	Mean (ng/L)	Med (ng/L)	Frequency	References
		Hong Kong River		0.59	0.24	0.25	92.31%	[29]
		Liaohe River Basins	ND	40.49	5.07		24.14%	[4]
		Surface Water		16.7		0.9		[38]
		Xinjiang Uygur Autonomous	0.85	14.85	2.92	1.53	100%	[1]
	Croatia	Sava	0.54	24.53	5.75	1.16	100%	[51]
Sarafloxacin	China	Dongting Lake	ND	7.94	5.51		23.5%	[21]
		Estuary		0.11				[23]
		Guangdong	ND	18.2		2.7	33.3%	[24]
		Hong Kong River		1.56	0.85	0.81	100%	[29]
		Liaohe River Basins	ND	6.02	0.36		6.9%	[4]
		Shatian Lake	ND	32	12.85	9	94.12%	[36]
		Surface Water		80.04		1.3		[38]
		Xiaoqing River (Mainstream)	LOQ	1892	16.5		71%	[46]
		Xiaoqing River (Tributaries)	LOQ	2290	17.8		60%	[46]
		Xinjiang Uygur Autonomous	0.36	2.35	0.98	0.78	100%	[1]
		Xiong'an New Area	ND	7.05			41%	[3]
		Yang River		0.67				[23]
		Yellow River (Mainstream)	LOQ	1899	17.7		72.7%	[46]
		Yellow River (Tributaries)	LOQ	1528	20.3		73%	[46]
	Croatia	Sava	0.49	2.79	1.05	0.70	100%	[51]
	Peru	Titicaca Lake	72.7	76.5	74.2		100%	[64]
Enoxacin	Brazilian	Surface Water	ND	386			5%	[85]
	China	Beibu Gulf	ND	2.95	1.24	0.85	94%	[10]
		Bohai Sea	ND	508	116			[16]

Continuation S2

Antibiotics	Country	Place	Min (ng/L)	Max (ng/L)	Mean (ng/L)	Med (ng/L)	Frequency	References
		Dongting Lake	ND	13.7	10.96		36.5%	[21]
		Surface Water		208	2.26			[38]
		Surface Water	0.2	508	71.7	48.6		[37]
		Surface Water in Basins	ND	92.8	21.5	1.6		[37]
		Xiaoqing River	ND	22.07	1.5			[44]
		Xiaoqing River (Mainstream)	LOQ	13428	448		82.3%	[46]
		Xiaoqing River (Tributaries)	13.9	18076	392		80%	[46]
		Xiong'an New Area	ND	2.5			77%	[3]
		Yangtze River (Nanjing)	ND	12.57	2			[81]
		Yellow River (Mainstream)	18.6	9185	323		78.8%	[46]
		Yellow River (Tributaries)	20.6	8070	319		71.4%	[46]
	France	Charmoise River	ND	1310	134			[16]
	Malaysia	Larut River	LOQ	2.55	0.14		11.11%	[62]
	Uganda	Victoria Lake	2.9	51		25	88%	[81]
Difloxacin	China	Beijing	ND	6.3		1.6	50%	[12]
		Changzhou	5.9	7.9		7.7	100%	[12]
		Dongting Lake	ND	4.75	2.38		45%	[21]
		Guangdong	0.84	1.24		0.85	100%	[24]
		Liaohe River Basins	ND	4.54	0.2		6.9%	[4]
		Surface Water		218.4		0.74		[38]
Levofloxacin	USA	Columbia River			1			[5]
		Sacramento River			2			[5]
	China	Chaohu Lake		89.86	＜25			[17]
		Surface Water		23.4		6		[38]

Continuation S2

Antibiotics	Country	Place	Min (ng/L)	Max (ng/L)	Mean (ng/L)	Med (ng/L)	Frequency	References
	South Africa	Apies River			2.4			[88]
	Uganda	Victoria Lake	1.8	29		12	96%	[81]
Moxifloxacin	USA	Sacramento River			0.012			[5]
	China	Liaohe River Basins	ND	41.1			13.79%	[4]
	China			300				[38]
	Spain		1.4	9.8			7%	[68]
Flumequine	China	Estuary		0.43				[23]
		Liaohe River Basins	ND	3.82	0.17		6.9%	[4]
		North South China		22.6				[53]
		Surface Water		137		0.83		[38]
		Xiaoqing River (Tributaries)	LOQ	20.8	LOQ (1.2)		43.1%	[46]
		Xiong'an New Area	ND	1.02			49%	[3]
		Yang River		0.39				[23]
		Yellow River (Tributaries)	LOQ	8.6	LOQ (1.2)		47.6%	[46]
	France	Surface Water		16				[25]
	Korea	Chungcheong Province		1.58				[53]
	Spain	NE Catalonia		8.9				[53]

Table S3. Toxicity of selected fluoroquinolone towards various trophic groups of organisms.

fluoroquinolone	group	organism	duration	EC50 (mg/L)	references
Ciprofloxacin	algae	*Anabaena flosaquae*	72h	0.01	[91]
		Chlorella sp.	72h	23	[92]
		Chlorella vulgaris	96h	31.07	[93]
		Chlorella vulgaris	96h	20.6	[94, 95]
		Desmodesmus subspicatus	72h	>8.04	[95]
		Desmodesmus subspicatus	72h	8.8	[95]
		Desmodesmus subspicatus	72h	8.04	[91]
		Microcysis panniformis	96h	0.01	[96]
		Microcystis aeruginosa	24h	0.017	[96, 97]
		Pseudokirchneriella subcapitata	72h	5.57	[95]
		Pseudokirchneriella subcapitata	72h	11.3	[95, 98]
		Pseudokirchneriella subcapitata	24h	18.7	[97]
		Pseudokirchneriella subcapitata	96h	4.83	[95]
		Pseudokirchneriella subcapitata	72h	6.7	[95]
		Pseudokirchneriella subcapitata	72h	2.97	[95]
		Raphidocelis subcapitata	72h	1.25	[99]
		Raphidocelis subcapitata	96h	4.61	[100]

		Raphidocelis subcapitata	72h	4.4	[100]
				3.1	[92]
	bacteria	*Cyanobacteria*		0.05	[92]
		Escherichia coli	24h	0.014	[101]
		Pseudomonas fluorescens	24h	0.12-0.49	[102]
		Pseudomonas putida	11d	0.08	[103]

Continuation S3

fluoroquinolone	group	organism	duration	EC50 (mg/L)	references
		Synechococus elongatus	96h	0.06	[100]
		Synechococus elongatus	72h	0.07	[100]
	crustacean	*Ceriodaphnia dubia*	21d	4	[104]
		Daphnia magna	48h	＞100	[6]
		Daphnia magna	21d	1.3-6.5(2.17)	[105]
		Daphnia magna	48h	28.59	[106]
		Daphnia magna	24h	＞192	[107]
		Daphnia magna	48h	60	[6]
		Daphnia magna		87.14	[6]
		Gammarus fossarum	7d	13.6	[108]
		Moina macrocopa	48h	71	[92]
	fish	*Danio rerio*		100	[6]
		Pimephales promelas	7d	＞10	[6]
				＞60	[92]
	plant	*Lemna minor*	24h	0.2	[97]
		Lemna minor	7d	0.63	[91]
Ofloxacin	algae	*Microcystis aeruginosa*	24h	0.02	[97]
		Pseudokirchneriella subcapitata	72h	1.44	[95]
		Pseudokirchneriella subcapitata	96h	4.74	[95]
		Pseudokirchneriella subcapitata	72h	12.1	[95, 97]
		Raphidocelis subcapitata	72h	4.18	[100]
		Raphidocelis subcapitata	96h	4.26	[100]
	bacteria	*Pseudomonas putida*	11d	0.01	[103]

Continuation S3

fluoroquinolone	group	organism	duration	EC50 (mg/L)	references
		Synechococus elongatus	72h	0.14	[100]
		Synechococus elongatus	96h	0.1	[107]
		Vibrio fischeri	24h	0.014	[109]
	crustacean	*Ceriodaphnia dubia*	7d	10	[6]
		Ceriodaphnia dubia	24h	17.41	[6]
		Ceriodaphnia dubia	48h	26.7	[6]
		Ceriodaphnia dubia	7d	3.13	[6]
		Daphnia magna	48h	76.58	[6]

		Daphnia magna	48h	31.75	[6]
		Daphnia magna	48h	17.41	[6]
	fish	*Danio rerio*	10d	＞16	[6]
		Pimephales promelas	7d	10	[6]
	mollusk	*Bellamya aeruginosa*	96h	149.3	[110]
		Bellamya aeruginosa	48h	222.6	[110]
	plant	*Lemna minor*	24h	0.13	[97]
Norfloxacin	algae	*Chlorella vulgaris*	72h	10.4	[95, 111]
		Desmodesmus subspicatus	72h	6.8	[95]
		Pseudokirchneriella subcapitata	72h	16.6	[95]
		Pseudokirchneriella subcapitata	72h	1.8	[95]
		Scenedesmus obliquus	96h	38.49	[112]
		Selenastrum capricornutum	72h	16.6	[111]
	bacteria	*Anabaena sp CPB4337*	72h	5.6	[109]
		Vibrio fischeri	15min	23.6	[109]

Continuation S3

fluoroquinolone	group	organism	duration	EC50 (mg/L)	references
	crustacean	*Ceriodaphnia dubia*	21d	4	[104]
	mollusk	*Bellamya aeruginosa*	48h	141.3	[110]
		Bellamya aeruginosa	96h	104.5	[110]
	plant	*Lactuca sativa*	5d	336	[113]
Enrofloxacin	algae	*Anabaena flosaquae*	72h	0.17	[97]
		Chlorella vulgaris	96h	0.12	[113]
		Desmodesmus subspicatus	3d	5.57	[91, 114]
		Microcystis aeruginosa	24h	0.05	[97, 114]
		Microcystis aeruginosa	72h	0.07-0.11(0.092)	[96, 97]
		Pseudokirchneriella subcapitata	3d	3.1	[114]
		Pseudokirchneriella subcapitata	24h	3.1	[97]
		Pseudokirchneriella subcapitata	96h	5.18	[115]
				2.97	[92, 97]
	bacteria	*Cyanobacteria*		0.01	[92]
	crustacean	*Ceriodaphnia dubia*	21d	5	[104]
		Daphnia magna	48h	16.72	[116]
		Daphnia magna	21d	24-36(30)	[105]
		Daphnia magna	48h	28.59	[106]
		Moina macrocopa	48h	71	[92]
		Moina macrocopa	48h	69	[92]
	fish			79.5	[92]
	plant	*Lemna minor*	7d	0.11	[91]
		Lemna minor	7d	0.114	[114]

Continuation S3

fluoroquinolone	group	organism	duration	EC50 (mg/L)	references
		Lemna minor	24h	0.11	[97]
		Lemna minor	7d	0.107	[114]
Lomefloxacin	algae	*Microcystis aeruginosa*	24h	0.19	[97]
	bacteria	*Vibrio fischeri*	24h	0.022	[109]
	plant	*Lemna minor*	24h	0.11	[97]
Marbofloxacin	crustacean	*Ceriodaphnia dubia*	21d	17	[104]
Enoxacin	bacteria	*Vibrio fischeri*	24h	0.049	[109]
Levofloxacin	algae	*Microcystis aeruginosa*	24h	0.008	[97]
	bacteria	*Streptococcus pneumoniae*	24h	3.57	[117]
	crustacean	*Ceriodaphnia dubia*	21d	11	[104]
		Daphnia magna	21d	6	[104]
		Daphnia magna	48h	＞40	[116]
	plant	*Lemna gibba*	14d	1.15	[118]
Moxifloxacin	algae	*Chorella sorokiniana*	96h	28.42	[119]
		Microcystis aeruginosa	96h	60.34	[120]
		Scenedesmus dimorphus	96h	26.37	[119]
	crustacean	*Ceriodaphnia dubia*	21d	26.00	[104]
		Daphnia magna	21d	7.00	[104]
	mollusk	*Glochidia*	24h	120.00	[121]
Flumequine	algae	*Dunaliella salina*	7d	18	[122]
		Microcystis aeruginosa	24h	1.96	[97]
		Microcystis aeruginosa	24h	＞8.8	[123]
		Pseudokirchneriella subcapitata	24h	5	[97]

Continuation S3

fluoroquinolone	group	organism	duration	EC50 (mg/L)	references
		Pseudokirchneriella subcapitata	24h	≈16	[123]
		Pseudokirchneriella subcapitata	96h	2.6	[124]
		Selenastrum capricornutum	7d	5	[122]
	bacteria	*Pseudomonas putida*	16h	0.82	[124]
		Vibrio fischeri	30min	12.10-15.34	[125]
		Vibrio fischeri	15min	11	[124]
	crustacean	*Daphnia magna*	48h	25.35	[116]
		Daphnia magna	24h	59	[124]
	mollusk	*Arbacia lixula*	72h	75.4	[126]
		Paracentrotus lividus	48h	31.01	[126]
	plant	*Lemna minor*	24h	2.47	[97]
		Lemna minor	7d	3	[124]

References

1. Wu S., Hua P., Gui D., Zhang J., Ying G..Krebs P. Occurrences, transport drivers, and risk assessments of antibiotics in typical oasis surface and groundwater. *Water Res,* **2022**, 225, 119138. http://doi.org/10.1016/j.watres.2022.119138

2. Yu X., Yu F., Li Z..Zhan J. Occurrence, distribution, and ecological risk assessment of pharmaceuticals and personal care products in the surface water of the middle and lower reaches of the Yellow River (Henan section). *J Hazard Mater,* **2023**, 443, 130369. http://doi.org/10.1016/j.jhazmat.2022.130369
3. Fu C., Xu B., Chen H., Zhao X., Li G., Zheng Y., Qiu W., Zheng C., Duan L..Wang W. Occurrence and distribution of antibiotics in groundwater, surface water, and sediment in Xiong'an New Area, China, and their relationship with antibiotic resistance genes. *Sci Total Environ,* **2022**, 807, 151011. http://doi.org/10.1016/j.scitotenv.2021.151011
4. Gao H., Zhao F., Li R., Jin S., Zhang H., Zhang K., Li S., Shu Q..Na G. Occurrence and distribution of antibiotics and antibiotic resistance genes in water of Liaohe River Basin, China. *Journal of Environmental Chemical Engineering,* **2022**, 10. http://doi.org/10.1016/j.jece.2022.108297
5. Mortimer M., Winchell A..Holden P.A. Evaluation of frameworks proposed as protective of antimicrobial resistance propagation in the environment. *Environ Int,* **2020**, 144, 106053. http://doi.org/10.1016/j.envint.2020.106053
6. Kovalakova P., Cizmas L., Mcdonald T.J., Marsalek B., Feng M..Sharma V.K. Occurrence and toxicity of antibiotics in the aquatic environment: A review. *Chemosphere,* **2020**, 251, 126351. http://doi.org/10.1016/j.chemosphere.2020.126351
7. Mastrangelo M.M., Valdes M.E., Eissa B., Ossana N.A., Barcelo D., Sabater S., Rodriguez-Mozaz S..Giorgi A.D.N. Occurrence and accumulation of pharmaceutical products in water and biota of urban lowland rivers. *Sci Total Environ,* **2022**, 828, 154303. http://doi.org/10.1016/j.scitotenv.2022.154303
8. Angeles L.F., Islam S., Aldstadt J., Saqeeb K.N., Alam M., Khan M.A., Johura F.T., Ahmed S.I..Aga D.S. Retrospective suspect screening reveals previously ignored antibiotics, antifungal compounds, and metabolites in Bangladesh surface waters. *Sci Total Environ,* **2020**, 712, 136285. http://doi.org/10.1016/j.scitotenv.2019.136285
9. Gomes M.P., Brito J.C.M., Vieira F., Kitamura R.S.A..Juneau P. Emerging Contaminants in Streams of Doce River Watershed, Minas Gerais, Brazil. *Frontiers in Environmental Science,* **2022**, 9. http://doi.org/10.3389/fenvs.2021.801599
10. Wu Q., Xiao S.K., Pan C.G., Yin C., Wang Y.H..Yu K.F. Occurrence, source apportionment and risk assessment of antibiotics in water and sediment from the subtropical Beibu Gulf, South China. *Sci Total Environ,* **2022**, 806, 150439. http://doi.org/10.1016/j.scitotenv.2021.150439
11. Su D., Ben W., Strobel B.W..Qiang Z. Impacts of wastewater treatment plant upgrades on the distribution and risks of pharmaceuticals in receiving rivers. *J Hazard Mater,* **2021**, 406, 124331. http://doi.org/10.1016/j.jhazmat.2020.124331
12. Ding G., Chen G., Liu Y., Li M..Liu X. Occurrence and risk assessment of fluoroquinolone antibiotics in reclaimed water and receiving groundwater with different replenishment pathways. *Sci Total Environ,* **2020**, 738, 139802. http://doi.org/10.1016/j.scitotenv.2020.139802
13. Zhang L., Bai J., Zhang K., Wang Y., Xiao R., Campos M., Acuna J..Jorquera M.A. Occurrence, bioaccumulation and ecological risks of antibiotics in the water-plant-sediment systems in different functional areas of the largest shallow lake in North China: Impacts of river input and historical agricultural activities. *Sci Total Environ,* **2023**, 857, 159260. http://doi.org/10.1016/j.scitotenv.2022.159260
14. Lu S., Lin C., Lei K., Xin M., Gu X., Lian M., Wang B., Liu X., Ouyang W..He M. Profiling of the spatiotemporal distribution, risks, and prioritization of antibiotics in the waters of Laizhou Bay, northern China. *J Hazard Mater,* **2022**, 424, 127487. http://doi.org/10.1016/j.jhazmat.2021.127487
15. Wang C., Lu Y., Wang C., Xiu C., Cao X., Zhang M..Song S. Distribution and ecological risks of pharmaceuticals and personal care products with different anthropogenic stresses in a coastal watershed of China. *Chemosphere,* **2022**, 303, 135176. http://doi.org/10.1016/j.chemosphere.2022.135176
16. Yang Q., Gao Y., Ke J., Show P.L., Ge Y., Liu Y., Guo R..Chen J. Antibiotics: An overview on the environmental occurrence, toxicity, degradation, and removal methods. *Bioengineered,* **2021**, 12, 7376-7416. http://doi.org/10.1080/21655979.2021.1974657
17. Zhang F., Yang L..Wang H. Co-occurrence characteristics of antibiotics and estrogens and their relationships in a lake system affected by wastewater. *J Environ Qual,* **2020**, 49, 1322-1333. http://doi.org/10.1002/jeq2.20128
18. Zhou Q., Liu G., Arif M., Shi X..Wang S. Occurrence and risk assessment of antibiotics in the surface water of Chaohu Lake and its tributaries in China. *Sci Total Environ,* **2022**, 807, 151040. http://doi.org/10.1016/j.scitotenv.2021.151040
19. Omuferen L.O., Maseko B..Olowoyo J.O. Occurrence of antibiotics in wastewater from hospital and convectional wastewater treatment plants and their impact on the effluent receiving rivers: current knowledge between 2010 and 2019. *Environ Monit Assess,* **2022**, 194, 306. http://doi.org/10.1007/s10661-022-09846-4
20. Guo X., Song R., Lu S., Liu X., Chen J., Wan Z..Bi B. Multi-Media Occurrence of Antibiotics and Antibiotic Resistance Genes in East Dongting Lake. *Frontiers in Environmental Science,* **2022**, 10. http://doi.org/10.3389/fenvs.2022.866332
21. Zhang B., Xu L., Hu Q., Zeng X..Yu Z. Occurrence, spatiotemporal distribution and potential ecological risks of antibiotics in Dongting Lake, China. *Environ Monit Assess,* **2020**, 192, 804. http://doi.org/10.1007/s10661-020-08761-w
22. Li J.Y., Wen J., Chen Y., Wang Q..Yin J. Antibiotics in cultured freshwater products in Eastern China: Occurrence, human health risks, sources, and bioaccumulation potential. *Chemosphere,* **2021**, 264, 128441. http://doi.org/10.1016/j.chemosphere.2020.128441
23. Liu K., Zhang D., Xiao X., Cui L..Zhang H. Occurrence of quinotone antibiotics and their impacts on aquatic environment in typical river-estuary system of Jiaozhou Bay, China. *Ecotoxicol Environ Saf,* **2020**, 190, 109993. http://doi.org/10.1016/j.ecoenv.2019.109993
24. Chen Y., Xie Q., Wan J., Yang S., Wang Y..Fan H. Occurrence and risk assessment of antibiotics in multifunctional reservoirs in Dongguan, China. *Environ Sci Pollut Res Int,* **2020**, 27, 13565-13574. http://doi.org/10.1007/s11356-019-07436-5
25. Huang L., Mo Y., Wu Z., Rad S., Song X., Zeng H., Bashir S., Kang B..Chen Z. Occurrence, distribution, and health risk assessment of quinolone antibiotics in water, sediment, and fish species of Qingshitan reservoir, South China. *Sci Rep,* **2020**, 10, 15777. http://doi.org/10.1038/s41598-020-72324-9
26. Chen J., Huang L., Wang Q., Zeng H., Xu J..Chen Z. Antibiotics in aquaculture ponds from Guilin, South of China: Occurrence, distribution, and health risk assessment. *Environ Res,* **2022**, 204, 112084. http://doi.org/10.1016/j.envres.2021.112084
27. Li X., Wang B., Liu F..Yu G. Occurrence and Removal of Pharmaceutical Contaminants in Urine: A Review. *Water,* **2023**, 15. http://doi.org/10.3390/w15081517
28. Wang C., Lu Y., Sun B., Zhang M., Wang R., Li X., Mao R., Cao Z..Song S. Contamination, transport, and ecological risks of pharmaceuticals and personal care products in a large irrigation region. *Sci Total Environ,* **2022**, 851, 158179. http://doi.org/10.1016/j.scitotenv.2022.158179
29. Liu J., Deng W.J., Ying G.G., Tsang E.P.K..Hong H.C. Occurrence and distribution of antibiotics in surface water. *Ecotoxicology,* **2022**, 31, 1111-1119. http://doi.org/10.1007/s10646-022-02567-x
30. Hu Y., Li L., Li B., Peng L., Xu Y., Zhou X., Li R..Song K. Spatial variations and ecological risks assessment of pharmaceuticals and personal care products (PPCPs) in typical lakes of Wuhan, China. *Process Safety and Environmental Protection,* **2023**, 174, 828-837. http://doi.org/10.1016/j.psep.2023.05.006

31. Maghsodian Z., Sanati A.M., Mashifana T., Sillanpaa M., Feng S., Nhat T..Ramavandi B. Occurrence and Distribution of Antibiotics in the Water, Sediment, and Biota of Freshwater and Marine Environments: A Review. *Antibiotics (Basel),* **2022**, 11. http://doi.org/10.3390/antibiotics11111461
32. Zhang G., Liu X., Lu S., Zhang J..Wang W. Occurrence of typical antibiotics in Nansi Lake's inflowing rivers and antibiotic source contribution to Nansi Lake based on principal component analysis-multiple linear regression model. *Chemosphere,* **2020**, 242, 125269. http://doi.org/10.1016/j.chemosphere.2019.125269
33. Shi J., Dong Y., Shi Y., Yin T., He W., An T., Tang Y., Hou X., Chong S., Chen D., Qin K..Lin H. Groundwater antibiotics and microplastics in a drinking-water source area, northern China: Occurrence, spatial distribution, risk assessment, and correlation. *Environ Res,* **2022**, 210, 112855. http://doi.org/10.1016/j.envres.2022.112855
34. Yin Z. Distribution and ecological risk assessment of typical antibiotics in the surface waters of seven major rivers, China. *Environ Sci Process Impacts,* **2021**, 23, 1088-1100. http://doi.org/10.1039/d1em00079a
35. Wang C., Zhao Y., Liu S., Xiao Q., Liang W..Song Y. Contamination, distribution, and risk assessment of antibiotics in the urban surface water of the Pearl River in Guangzhou, South China. *Environ Monit Assess,* **2021**, 193, 98. http://doi.org/10.1007/s10661-021-08887-5
36. Yang H., Xu M., Wang L., Wang X., Jeppesen E..Zhang W. Metagenomic analysis to determine the characteristics of antibiotic resistance genes in typical antibiotic-contaminated sediments. *J Environ Sci (China),* **2023**, 128, 12-25. http://doi.org/10.1016/j.jes.2022.08.014
37. Huang F., An Z., Moran M.J..Liu F. Recognition of typical antibiotic residues in environmental media related to groundwater in China (2009-2019). *J Hazard Mater,* **2020**, 399, 122813. http://doi.org/10.1016/j.jhazmat.2020.122813
38. Li Z., Li M., Zhang Z., Li P., Zang Y..Liu X. Antibiotics in aquatic environments of China: A review and meta-analysis. *Ecotoxicol Environ Saf,* **2020**, 199, 110668. http://doi.org/10.1016/j.ecoenv.2020.110668
39. Chen C.X., Aris A., Yong E.L..Noor Z.Z. Evaluation of the occurrence of antibiotics at different treatment stages of decentralised and conventional sewage treatment plants. *International Journal of Environmental Science and Technology,* **2021**, 19, 5547-5562. http://doi.org/10.1007/s13762-021-03519-4
40. Yang J., Huang Y., Chen Y., Hassan M., Zhang X., Zhang B., Gin K.Y..He Y. Multi-phase distribution, spatiotemporal variation and risk assessment of antibiotics in a typical urban-rural watershed. *Ecotoxicol Environ Saf,* **2020**, 206, 111156. http://doi.org/10.1016/j.ecoenv.2020.111156
41. Wu Y., Song S., Chen X., Shi Y., Cui H., Liu Y..Yang S. Source-specific ecological risks and critical source identification of PPCPs in surface water: Comparing urban and rural areas. *Sci Total Environ,* **2023**, 854, 158792. http://doi.org/10.1016/j.scitotenv.2022.158792
42. Ramírez-Morales D., Masís-Mora M., Montiel-Mora J.R., Cambronero-Heinrichs J.C., Pérez-Rojas G., Tormo-Budowski R., Méndez-Rivera M., Briceño-Guevara S., Gutiérrez-Quirós J.A., Arias-Mora V., Brenes-Alfaro L., Beita-Sandí W..Rodríguez-Rodríguez C.E. Multi-residue analysis of pharmaceuticals in water samples by liquid chromatography- mass spectrometry: Quality assessment and application to the risk assessment of urban-influenced surface waters in a metropolitan area of Central America. *Process Safety and Environmental Protection,* **2021**, 153, 289-300. http://doi.org/10.1016/j.psep.2021.07.025
43. Wang R., Ji M., Zhai H., Guo Y..Liu Y. Occurrence of antibiotics and antibiotic resistance genes in WWTP effluent-receiving water bodies and reclaimed wastewater treatment plants. *Sci Total Environ,* **2021**, 796, 148919. http://doi.org/10.1016/j.scitotenv.2021.148919
44. Ci M., Zhang G., Yan X., Dong W., Xu W., Wang W..Fan Y. Occurrence of antibiotics in the Xiaoqing River basin and antibiotic source contribution-a case study of Jinan city, China. *Environ Sci Pollut Res Int,* **2021**, 28, 25241-25254. http://doi.org/10.1007/s11356-020-12202-z
45. Li J., Cui M..Zhang H. Spatial and temporal variations of antibiotics in a tidal river. *Environ Monit Assess,* **2020**, 192, 336. http://doi.org/10.1007/s10661-020-08313-2
46. Wang K., Su Z., Reguyal F., Bian R., Li W., Yu H., Sun Y., Zhuang Y..Shang W. Seasonal occurrence of multiple classes of antibiotics in East China rivers and their association with suspended particulate matter. *Sci Total Environ,* **2022**, 853, 158579. http://doi.org/10.1016/j.scitotenv.2022.158579
47. He P., Wu J., Peng J., Wei L., Zhang L., Zhou Q..Wu Z. Pharmaceuticals in drinking water sources and tap water in a city in the middle reaches of the Yangtze River: occurrence, spatiotemporal distribution, and risk assessment. *Environ Sci Pollut Res Int,* **2022**, 29, 2365-2374. http://doi.org/10.1007/s11356-021-15363-7
48. Zhang G., Lu S., Wang Y., Liu X., Liu Y., Xu J., Zhang T., Wang Z..Yang Y. Occurrence of antibiotics and antibiotic resistance genes and their correlations in lower Yangtze River, China. *Environ Pollut,* **2020**, 257, 113365. http://doi.org/10.1016/j.envpol.2019.113365
49. Feng J., Liu Q., Ru X., Xi N..Sun J. Occurrence and distribution of priority pharmaceuticals in the Yellow River and the Huai River in Henan, China. *Environ Sci Pollut Res Int,* **2020**, 27, 16816-16826. http://doi.org/10.1007/s11356-020-08131-6
50. Sun J., Ke Z., Zhang Y., Wu Q., Chen Y..Tang J. Pharmaceutical active compounds in a heavily industrialized and urbanized bay, Eastern China. *Environ Sci Pollut Res Int,* **2023**, 30, 51624-51637. http://doi.org/10.1007/s11356-023-26019-z
51. Malev O., Babic S., Sima Cota A., Stipanicev D., Repec S., Drnic M., Lovric M., Bojanic K., Radic Brkanac S., Coz-Rakovac R..Klobucar G. Combining short-term bioassays using fish and crustacean model organisms with ToxCast in vitro data and broad-spectrum chemical analysis for environmental risk assessment of the river water (Sava, Croatia). *Environ Pollut,* **2022**, 292, 118440. http://doi.org/10.1016/j.envpol.2021.118440
52. Rodriguez-Mozaz S., Vaz-Moreira I., Varela Della Giustina S., Llorca M., Barcelo D., Schubert S., Berendonk T.U., Michael-Kordatou I., Fatta-Kassinos D., Martinez J.L., Elpers C., Henriques I., Jaeger T., Schwartz T., Paulshus E., O'sullivan K., Parnanen K.M.M., Virta M., Do T.T., Walsh F..Manaia C.M. Antibiotic residues in final effluents of European wastewater treatment plants and their impact on the aquatic environment. *Environ Int,* **2020**, 140, 105733. http://doi.org/10.1016/j.envint.2020.105733
53. Zainab S.M., Junaid M., Xu N..Malik R.N. Antibiotics and antibiotic resistant genes (ARGs) in groundwater: A global review on dissemination, sources, interactions, environmental and human health risks. *Water Res,* **2020**, 187, 116455. http://doi.org/10.1016/j.watres.2020.116455
54. Miranda C.D., Concha C., Godoy F.A..Lee M.R. Aquatic Environments as Hotspots of Transferable Low-Level Quinolone Resistance and Their Potential Contribution to High-Level Quinolone Resistance. *Antibiotics (Basel),* **2022**, 11. http://doi.org/10.3390/antibiotics11111487
55. Arun S., Xin L., Gaonkar O., Neppolian B., Zhang G..Chakraborty P. Antibiotics in sewage treatment plants, receiving water bodies and groundwater of Chennai city and the suburb, South India: Occurrence, removal efficiencies, and risk assessment. *Sci Total Environ,* **2022**, 851, 158195. http://doi.org/10.1016/j.scitotenv.2022.158195
56. Singh V..Suthar S. Occurrence, seasonal variations, and ecological risk of pharmaceuticals and personal care products in River Ganges at two holy cities of India. *Chemosphere,* **2021**, 268, 129331. http://doi.org/10.1016/j.chemosphere.2020.129331

57. O'flynn D., Lawler J., Yusuf A., Parle-Mcdermott A., Harold D., Mc Cloughlin T., Holland L., Regan F..White B. A review of pharmaceutical occurrence and pathways in the aquatic environment in the context of a changing climate and the COVID-19 pandemic. *Anal Methods,* **2021**, 13, 575-594. http://doi.org/10.1039/d0ay02098b
58. Muriuki C.W., Home P.G., Raude J.M., Ngumba E.K., Munala G.K., Kairigo P.K., Gachanja A.N..Tuhkanen T.A. Occurrence, distribution, and risk assessment of pharmerciuticals in wastewater and open surface drains of peri-urban areas: Case study of Juja town, Kenya. *Environ Pollut,* **2020**, 267, 115503. http://doi.org/10.1016/j.envpol.2020.115503
59. K'oreje K., Okoth M., Van Langenhove H..Demeestere K. Occurrence and point-of-use treatment of contaminants of emerging concern in groundwater of the Nzoia River basin, Kenya. *Environ Pollut,* **2022**, 297, 118725. http://doi.org/10.1016/j.envpol.2021.118725
60. Kairigo P., Ngumba E., Sundberg L.-R., Gachanja A..Tuhkanen T. Contamination of Surface Water and River Sediments by Antibiotic and Antiretroviral Drug Cocktails in Low and Middle-Income Countries: Occurrence, Risk and Mitigation Strategies. *Water,* **2020**, 12. http://doi.org/10.3390/w12051376
61. Khairul Hasni N.A., Anual Z.F., Rashid S.A., Syed Abu Thahir S., Veloo Y., Fang K.S..Mazeli M.I. Occurrence of endocrine disruptors in Malaysia's water systems: A scoping review. *Environ Pollut,* **2023**, 324, 121095. http://doi.org/10.1016/j.envpol.2023.121095
62. Low K., Chai L., Lee C., Zhang G., Zhang R., Vahab V..Bong C. Prevalence and risk assessment of antibiotics in riverine estuarine waters of Larut and Sangga Besar River, Perak. *Journal of Oceanology and Limnology,* **2020**, 39, 122-134. http://doi.org/10.1007/s00343-020-9246-y
63. Truong T., Hoang T.L., Tran L.T., Pham T.P.T..Le T.-H. Prevalence of Antibiotic Resistance Genes in the Saigon River Impacted by Anthropogenic Activities. *Water,* **2021**, 13. http://doi.org/10.3390/w13162234
64. Vilca F.Z., Galarza N.C., Tejedo J.R., Cuba W.a.Z., Quiróz C.N.C..Tornisielo V.L. Occurrence of residues of veterinary antibiotics in water, sediment and trout tissue (Oncorhynchus mykiss) in the southern area of Lake Titicaca, Peru. *Journal of Great Lakes Research,* **2021**, 47, 1219-1227. http://doi.org/10.1016/j.jglr.2021.04.012
65. Giebultowicz J., Nalecz-Jawecki G., Harnisz M., Kucharski D., Korzeniewska E..Plaza G. Environmental Risk and Risk of Resistance Selection Due to Antimicrobials' Occurrence in Two Polish Wastewater Treatment Plants and Receiving Surface Water. *Molecules,* **2020**, 25. http://doi.org/10.3390/molecules25061470
66. Serra-Compte A., Pikkemaat M.G., Elferink A., Almeida D., Diogene J., Campillo J.A., Llorca M., Alvarez-Munoz D., Barcelo D..Rodriguez-Mozaz S. Combining an effect-based methodology with chemical analysis for antibiotics determination in wastewater and receiving freshwater and marine environment. *Environ Pollut,* **2021**, 271, 116313. http://doi.org/10.1016/j.envpol.2020.116313
67. Fonseca E., Hernandez F., Ibanez M., Rico A., Pitarch E..Bijlsma L. Occurrence and ecological risks of pharmaceuticals in a Mediterranean river in Eastern Spain. *Environ Int,* **2020**, 144, 106004. http://doi.org/10.1016/j.envint.2020.106004
68. Gozzo S., Moles S., Kińska K., Ormad M.P., Mosteo R., Gómez J., Laborda F..Szpunar J. Screening for Antibiotics and Their Degradation Products in Surface and Wastewaters of the POCTEFA Territory by Solid-Phase Extraction-UPLC-Electrospray MS/MS. *Water,* **2022**, 15. http://doi.org/10.3390/w15010014
69. Golovko O., Orn S., Sorengard M., Frieberg K., Nassazzi W., Lai F.Y..Ahrens L. Occurrence and removal of chemicals of emerging concern in wastewater treatment plants and their impact on receiving water systems. *Sci Total Environ,* **2021**, 754, 142122. http://doi.org/10.1016/j.scitotenv.2020.142122
70. Nantaba F., Wasswa J., Kylin H., Palm W.U., Bouwman H..Kummerer K. Occurrence, distribution, and ecotoxicological risk assessment of selected pharmaceutical compounds in water from Lake Victoria, Uganda. *Chemosphere,* **2020**, 239, 124642. http://doi.org/10.1016/j.chemosphere.2019.124642
71. Dalahmeh S., Bjornberg E., Elenstrom A.K., Niwagaba C.B..Komakech A.J. Pharmaceutical pollution of water resources in Nakivubo wetlands and Lake Victoria, Kampala, Uganda. *Sci Total Environ,* **2020**, 710, 136347. http://doi.org/10.1016/j.scitotenv.2019.136347
72. Da Le N., Hoang A.Q., Hoang T.T.H., Nguyen T.a.H., Duong T.T., Pham T.M.H., Nguyen T.D., Hoang V.C., Phung T.X.B., Le H.T., Tran C.S., Dang T.H., Vu N.T., Nguyen T.N..Le T.P.Q. Antibiotic and antiparasitic residues in surface water of urban rivers in the Red River Delta (Hanoi, Vietnam): concentrations, profiles, source estimation, and risk assessment. *Environ Sci Pollut Res Int,* **2021**, 28, 10622-10632. http://doi.org/10.1007/s11356-020-11329-3
73. Wang L., Li H., Dang J., Guo H., Zhu Y..Han W. Occurrence, distribution, and partitioning of antibiotics in surface water and sediment in a typical tributary of Yellow River, China. *Environ Sci Pollut Res Int,* **2021**, 28, 28207-28221. http://doi.org/10.1007/s11356-021-12634-1
74. Zhao B., Xu J., Zhang G., Lu S., Liu X., Li L..Li M. Occurrence of antibiotics and antibiotic resistance genes in the Fuxian Lake and antibiotic source analysis based on principal component analysis-multiple linear regression model. *Chemosphere,* **2021**, 262, 127741. http://doi.org/10.1016/j.chemosphere.2020.127741
75. Peng Q., Song J., Li X., Yuan H., Liu M., Duan L..Zuo J. Pharmaceutically active compounds (PhACs) in surface sediments of the Jiaozhou Bay, north China. *Environ Pollut,* **2020**, 266, 115245. http://doi.org/10.1016/j.envpol.2020.115245
76. Dong J., Yan D., Mo K., Chen Q., Zhang J., Chen Y..Wang Z. Antibiotics along an alpine river and in the receiving lake with a catchment dominated by grazing husbandry. *J Environ Sci (China),* **2022**, 115, 374-382. http://doi.org/10.1016/j.jes.2021.08.007
77. Liu Y., Cai D., Li X., Wu Q., Ding P., Shen L., Yang J., Hu G., Wu J..Zhang L. Occurrence, fate, and risk assessment of antibiotics in typical pharmaceutical manufactories and receiving water bodies from different regions. *PLoS One,* **2023**, 18, e0270945. http://doi.org/10.1371/journal.pone.0270945
78. Ding J., Jiang H., Wu X., Zhang S., Mamitiana Razanajatovo R..Zou H. Investigation and assessment of environmental pollution in Gonghu Bay, Taihu Lake, China: A year-long study. *Human and Ecological Risk Assessment: An International Journal,* **2019**, 26, 906-920. http://doi.org/10.1080/10807039.2018.1545565
79. Yuntao Z., Jiang L., Tianyu W., Kai M., Zhentao C., Qianwen Y., Yongheng D., Yu C., Bin W..Xia H. Characteristics of antibiotic resistance genes and microbial community distribution in Wanfeng Lake, upper Pearl River, China. *Environmental Science and Pollution Research,* **2023**.
80. Omuferen L.O., Maseko B..Olowoyo J.O. Occurrence of antibiotics in wastewater from hospital and convectional wastewater treatment plants and their impact on the effluent receiving rivers: current knowledge between 2010 and 2019. *Environmental Monitoring and Assessment,* **2022**, 194, -.
81. Liu Y., Chen Y., Feng M., Chen J., Shen W..Zhang S. Occurrence of antibiotics and antibiotic resistance genes and their correlations in river-type drinking water source, China. *Environ Sci Pollut Res Int,* **2021**, 28, 42339-42352. http://doi.org/10.1007/s11356-021-13637-8
82. Lin H., Huang L., Gao Z., Lin W..Ren Y. Comparative analysis of the removal and transformation of 10 typical pharmaceutical and personal care products in secondary treatment of sewage: A case study of two biological treatment processes. *Journal of Environmental Chemical Engineering,* **2022**, 10. http://doi.org/10.1016/j.jece.2022.107638

83. Hanna N., Purohit M., Diwan V., Chandran S.P., Riggi E., Parashar V., Tamhankar A.J..Lundborg C.S. Monitoring of Water Quality, Antibiotic Residues, and Antibiotic-Resistant Escherichiacoli in the Kshipra River in India over a 3-Year Period. *Int J Environ Res Public Health,* **2020**, 17. http://doi.org/10.3390/ijerph17217706
84. Lenart-Boron A., Prajsnar J., Guzik M., Boron P..Chmiel M. How much of antibiotics can enter surface water with treated wastewater and how it affects the resistance of waterborne bacteria: A case study of the Bialka river sewage treatment plant. *Environ Res,* **2020**, 191, 110037. http://doi.org/10.1016/j.envres.2020.110037
85. Reis E.O., Santos L.V.S..Lange L.C. Prioritization and environmental risk assessment of pharmaceuticals mixtures from Brazilian surface waters. *Environ Pollut,* **2021**, 288, 117803. http://doi.org/10.1016/j.envpol.2021.117803
86. Lee S., Kim C., Liu X., Lee S., Kho Y., Kim W.K., Kim P..Choi K. Ecological Risk Assessment of Amoxicillin, Enrofloxacin, and Neomycin: Are Their Current Levels in the Freshwater Environment Safe? *Toxics,* **2021**, 9. http://doi.org/10.3390/toxics9080196
87. Mai Z., Xiong X., Hu H., Jia J., Wu C..Wang G. Occurrence, distribution, and ecological risks of antibiotics in Honghu Lake and surrounding aquaculture ponds, China. *Environ Sci Pollut Res Int,* **2023**, 30, 50732-50742. http://doi.org/10.1007/s11356-023-25931-8
88. Mashile G.P. Pharmaceuticals and personal care products in wastewater, river water and tap water: occurrences and removal [M]. University of Johannesburg (South Africa), 2020.
89. Gray A.D., Todd D..Hershey A.E. The seasonal distribution and concentration of antibiotics in rural streams and drinking wells in the piedmont of North Carolina. *Sci Total Environ,* **2020**, 710, 136286. http://doi.org/10.1016/j.scitotenv.2019.136286
90. Gray A. The use of non-target high-resolution mass spectrometry screening to detect the presence of antibiotic residues in urban streams of Greensboro North Carolina. *J Environ Health Sci Eng,* **2021**, 19, 1313-1321. http://doi.org/10.1007/s40201-021-00688-9
91. Ebert I., Bachmann J., Kuhnen U., Kuster A., Kussatz C., Maletzki D..Schluter C. Toxicity of the fluoroquinolone antibiotics enrofloxacin and ciprofloxacin to photoautotrophic aquatic organisms. *Environ Toxicol Chem,* **2011**, 30, 2786-2792. http://doi.org/10.1002/etc.678
92. Andrieu M., Rico A., Phu T.M., Huong D.T.T., Phuong N.T..Van Den Brink P.J. Ecological risk assessment of the antibiotic enrofloxacin applied to Pangasius catfish farms in the Mekong Delta, Vietnam. *Chemosphere,* **2015**, 119, 407-414. http://doi.org/10.1016/j.chemosphere.2014.06.062
93. Wu M.N., Wang X.C..Ma X.Y. Phytotoxicity comparison of organic contaminants and heavy metals using *Chlorella vulgaris*. *Desalination and Water Treatment,* **2015**, 1-8. http://doi.org/10.1080/19443994.2015.1110537
94. Xiangping N. RESPONSE OF THE FRESHWATER ALGA CHLORELLA VULGARIS TO TRICHLOROISOCYANURIC ACID AND CIPROFLOXACIN(Abstract); proceedings of the American Chemical Society National Meeting, F, 2007 [C].
95. Zhu L., Santiago-Schubel B., Xiao H., Hollert H..Kueppers S. Electrochemical oxidation of fluoroquinolone antibiotics: Mechanism, residual antibacterial activity and toxicity change. *Water Res,* **2016**, 102, 52-62. http://doi.org/10.1016/j.watres.2016.06.005
96. Azevedo F.C.R., Vaz I.C.D., Barbosa F.a.R..Magalhães S.M.S. Toxicological effects of ciprofloxacin and chlorhexidine on growth and chlorophyll a synthesis of freshwater cyanobacteria. *Brazilian Journal of Pharmaceutical Sciences,* **2019**, 55. http://doi.org/10.1590/s2175-97902019000217661
97. April A. Robinson J.B.B..Lydy M.J. TOXICITY OF FLUOROQUINOLONE ANTIBIOTICS TO AQUATIC ORGANISMS. *Environmental Toxicology and Chemistry,* **2005**, 24, p.423-430.
98. Magdaleno A., Saenz M.E., Juarez A.B..Moretton J. Effects of six antibiotics and their binary mixtures on growth of *Pseudokirchneriella subcapitata*. *Ecotoxicol Environ Saf,* **2015**, 113, 72-78. http://doi.org/10.1016/j.ecoenv.2014.11.021
99. Jafari-Nodoushan H., Fazeli M.R., Faramarzi M.A..Samadi N. Hierarchically-structured laccase@$Ni_3(PO_4)_2$ hybrid nanoflowers for antibiotic degradation: Application in real wastewater effluent and toxicity evaluation. *Int J Biol Macromol,* **2023**, 234, 123574. http://doi.org/10.1016/j.ijbiomac.2023.123574
100. Kovalakova P., Cizmas L., Feng M., Mcdonald T.J., Marsalek B..Sharma V.K. Oxidation of antibiotics by ferrate(VI) in water: Evaluation of their removal efficiency and toxicity changes. *Chemosphere,* **2021**, 277, 130365. http://doi.org/10.1016/j.chemosphere.2021.130365
101. Carneiro R.B., Pozzi E., Corbi J.J..Zaiat M. Ecotoxicity and Antimicrobial Inhibition Assessment of Effluent from an Anaerobic Bioreactor Applied to the Removal of Sulfamethoxazole and Ciprofloxacin Antibiotics from Domestic Sewage. *Water, Air, & Soil Pollution,* **2021**, 232, 1-13.
102. Danner M.C., Azams S.O., Robertson A., Perkins D., Behrends V..Reiss J. It More than Adds Up: Interaction of Antibiotic Mixing and Temperature. *Life (Basel),* **2021**, 11. http://doi.org/10.3390/life11121435
103. Nakata H., Kannan K., Jones P.D..Giesy J.P. Determination of fluoroquinolone antibiotics in wastewater effluents by liquid chromatography-mass spectrometry and fluorescence detection. *Chemosphere,* **2005**, 58, 759-766. http://doi.org/10.1016/j.chemosphere.2004.08.097
104. Kergaravat S.V., Hernandez S.R..Gagneten A.M. Second-, third- and fourth-generation quinolones: Ecotoxicity effects on Daphnia and Ceriodaphnia species. *Chemosphere,* **2021**, 262, 127823. http://doi.org/10.1016/j.chemosphere.2020.127823
105. Dalla Bona M., Zounkova R., Merlanti R., Blaha L..De Liguoro M. Effects of enrofloxacin, ciprofloxacin, and trimethoprim on two generations of *Daphnia magna*. *Ecotoxicol Environ Saf,* **2015**, 113, 152-158. http://doi.org/10.1016/j.ecoenv.2014.11.018
106. Bawa-Allah K.A..Ehimiyein A.O. Ecotoxicological effects of human and veterinary antibiotics on water flea (*Daphnia magna*). *Environ Toxicol Pharmacol,* **2022**, 94, 103932. http://doi.org/10.1016/j.etap.2022.103932
107. Dave G..Herger G. Determination of detoxification to *Daphnia magna* of four pharmaceuticals and seven surfactants by activated sludge. *Chemosphere,* **2012**, 88, 459-466. http://doi.org/10.1016/j.chemosphere.2012.02.070
108. Konschak M., Zubrod J.P., Baudy P., Fink P., Kenngott K., Luderwald S., Englert K., Jusi C., Schulz R..Bundschuh M. The importance of diet-related effects of the antibiotic ciprofloxacin on the leaf-shredding invertebrate Gammarus fossarum (*Crustacea; Amphipoda*). *Aquat Toxicol,* **2020**, 222, 105461. http://doi.org/10.1016/j.aquatox.2020.105461
109. Menz J., Baginska E., Arrhenius A., Haiss A., Backhaus T..Kummerer K. Antimicrobial activity of pharmaceutical cocktails in sewage treatment plant effluent - An experimental and predictive approach to mixture risk assessment. *Environ Pollut,* **2017**, 231, 1507-1517. http://doi.org/10.1016/j.envpol.2017.09.009
110. He S., Sun Q., Liu Y., Li L..Yang X. Acute Toxicity, Bioaccumulation, and Bioreduction of Two Antibiotics, Norfloxacin and Ofloxacin, in the Snail *Bellamya aeruginosa*. *Water, Air, & Soil Pollution,* **2020**, 231. http://doi.org/10.1007/s11270-020-4458-5
111. Eguchi K., Nagase H., Ozawa M., Endoh Y.S., Goto K., Hirata K., Miyamoto K..Yoshimura H. Evaluation of antimicrobial agents for veterinary use in the ecotoxicity test using microalgae. *Chemosphere,* **2004**, 57, 1733-1738. http://doi.org/10.1016/j.chemosphere.2004.07.017
112. Nie X., Gu J., Lu J., Pan W..Yang Y. Effects of norfloxacin and butylated hydroxyanisole on the freshwater microalga *Scenedesmus obliquus*. *Ecotoxicology,* **2009**, 18, 677-684. http://doi.org/10.1007/s10646-009-0334-1

113. Montanes M.T., Garcia-Gabaldon M., Roca-Perez L., Giner-Sanz J.J., Mora-Gomez J..Perez-Herranz V. Analysis of norfloxacin ecotoxicity and the relation with its degradation by means of electrochemical oxidation using different anodes. *Ecotoxicol Environ Saf,* **2020**, 188, 109923. http://doi.org/10.1016/j.ecoenv.2019.109923
114. Rico A., Zhao W., Gillissen F., Lurling M..Van Den Brink P.J. Effects of temperature, genetic variation and species competition on the sensitivity of algae populations to the antibiotic enrofloxacin. *Ecotoxicol Environ Saf,* **2018**, 148, 228-236. http://doi.org/10.1016/j.ecoenv.2017.10.010
115. Magdaleno A., Carusso S..Moretton J. Toxicity and Genotoxicity of Three Antimicrobials Commonly Used in Veterinary Medicine. *Bull Environ Contam Toxicol,* **2017**, 99, 315-320. http://doi.org/10.1007/s00128-017-2091-9
116. Tolosi R..De Liguoro M. Delayed toxicity of three fluoroquinolones and their mixtures after neonatal or embryonic exposure, in *Daphnia magna*. *Ecotoxicol Environ Saf,* **2021**, 225, 112778. http://doi.org/10.1016/j.ecoenv.2021.112778
117. Tasso L., De Andrade C..Dalla Costa T. Pharmacokinetic/pharmacodynamic modelling of the bactericidal activity of free lung concentrations of levofloxacin and gatifloxacin against Streptococcus pneumoniae. *Int J Antimicrob Agents,* **2011**, 38, 307-313. http://doi.org/10.1016/j.ijantimicag.2011.05.015
118. Brain R.A., Johnson D.J., Richards S.M., Hanson M.L., Sanderson H., Lam M.W., Young C., Mabury S.A., Sibley P.K..Solomon K.R. Microcosm evaluation of the effects of an eight pharmaceutical mixture to the aquatic macrophytes *Lemna gibba* and *Myriophyllum sibiricum*. *Aquat Toxicol,* **2004**, 70, 23-40. http://doi.org/10.1016/j.aquatox.2004.06.011
119. Li Z., Gao X., Bao J., Li S., Wang X., Li Z..Zhu L. Evaluation of growth and antioxidant responses of freshwater microalgae *Chlorella sorokiniana* and *Scenedesmus dimorphus* under exposure of moxifloxacin. *Sci Total Environ,* **2023**, 858, 159788. http://doi.org/10.1016/j.scitotenv.2022.159788
120. Wan L., Wu Y., Zhang B., Yang W., Ding H..Zhang W. Effects of moxifloxacin and gatifloxacin stress on growth, photosynthesis, antioxidant responses, and microcystin release in *Microcystis aeruginosa*. *J Hazard Mater,* **2021**, 409, 124518. http://doi.org/10.1016/j.jhazmat.2020.124518
121. Gilroy E.A., Klinck J.S., Campbell S.D., Mcinnis R., Gillis P.L..De Solla S.R. Toxicity and bioconcentration of the pharmaceuticals moxifloxacin, rosuvastatin, and drospirenone to the unionid mussel *Lampsilis siliquoidea*. *Sci Total Environ,* **2014**, 487, 537-544. http://doi.org/10.1016/j.scitotenv.2014.03.051
122. De Orte M.R., Carballeira C., Viana I.G..Carballeira A. Assessing the toxicity of chemical compounds associated with marine land-based fish farms: The use of mini-scale microalgal toxicity tests. *Chemistry and Ecology,* **2013**, 29, 554-563. http://doi.org/10.1080/02757540.2013.790381
123. Van Der Grinten E., Pikkemaat M.G., Van Den Brandhof E.J., Stroomberg G.J..Kraak M.H. Comparing the sensitivity of algal, cyanobacterial and bacterial bioassays to different groups of antibiotics. *Chemosphere,* **2010**, 80, 1-6. http://doi.org/10.1016/j.chemosphere.2010.04.011
124. Zounkova R., Klimesova Z., Nepejchalova L., Hilscherova K..Blaha L. Complex evaluation of ecotoxicity and genotoxicity of antimicrobials oxytetracycline and flumequine used in aquaculture. *Environ Toxicol Chem,* **2011**, 30, 1184-1189. http://doi.org/10.1002/etc.486
125. Lalumera G.M., Calamari D., Galli P., Castiglioni S., Crosa G..Fanelli R. Preliminary investigation on the environmental occurrence and effects of antibiotics used in aquaculture in Italy. *Chemosphere,* **2004**, 54, 661-668. http://doi.org/10.1016/j.chemosphere.2003.08.001
126. Carballeira C., De Orte M.R., Viana I.G., Delvalls T.A..Carballeira A. Assessing the toxicity of chemical compounds associated with land-based marine fish farms: the sea urchin embryo bioassay with *Paracentrotus lividus* and *Arbacia lixula*. *Arch Environ Contam Toxicol,* **2012**, 63, 249-261. http://doi.org/10.1007/s00244-012-9769-0
